# Net rate of lateral gene transfer in marine prokaryoplankton

**DOI:** 10.1093/ismejo/wraf159

**Published:** 2025-09-05

**Authors:** Ramunas Stepanauskas, Julia M Brown, Shayesteh Arasti, Uyen Mai, Gregory Gavelis, Maria Pachiadaki, Oliver Bezuidt, Jacob H Munson-McGee, Tianyi Chang, Steven J Biller, Paul M Berube, Siavash Mirarab

**Affiliations:** Bigelow Laboratory for Ocean Sciences; East Boothbay, ME 04544, United States; Bigelow Laboratory for Ocean Sciences; East Boothbay, ME 04544, United States; Department of Computer Science and Engineering, University of California; San Diego, La Jolla, CA 92093, United States; Department of Computer Science and Engineering, University of California; San Diego, La Jolla, CA 92093, United States; Bigelow Laboratory for Ocean Sciences; East Boothbay, ME 04544, United States; Woods Hole Oceanographic Institution; Woods Hole, MA 02543, United States; Bigelow Laboratory for Ocean Sciences; East Boothbay, ME 04544, United States; Department of Biochemistry, Genetics and Microbiology, University of Pretoria, Pretoria 0028, South Africa; Bigelow Laboratory for Ocean Sciences; East Boothbay, ME 04544, United States; Bigelow Laboratory for Ocean Sciences; East Boothbay, ME 04544, United States; Department of Biological Sciences, Wellesley College; Wellesley, MA 02481, United States; Department of Civil and Environmental Engineering, Massachusetts Institute of Technology; Cambridge, MA 02139, United States; Department of Electrical and Computer Engineering, University of California; San Diego, La Jolla, CA 92093, United States

**Keywords:** lateral gene transfer, microbial evolution, bacteria, plankton, ocean, marine, epipelagic, single-cell genomics

## Abstract

Lateral gene transfer is a major evolutionary process in Bacteria and Archaea. Despite its importance, lateral gene transfer quantification in nature using traditional phylogenetic methods has been hampered by the rarity of most genes within the enormous microbial pangenomes. Here, we estimated lateral gene transfer rates within the epipelagic tropical and subtropical ocean using a global, randomized collection of single amplified genomes and a non-phylogenetic computational approach. By comparing the fraction of shared genes between pairs of genomes against a lateral gene transfer-free model, we show that an average cell line laterally acquires and retains ~13% of its genes every 1 million years. This translates to a net lateral gene transfer rate of ~250 genes L^−1^ seawater day^−1^ and involves both “flexible” and “core” genes. Our study indicates that whereas most genes are exchanged among closely related cells, the range of lateral gene transfer exceeds the contemporary definition of bacterial species, thus providing prokaryoplankton with extensive genetic resources for lateral gene transfer-based adaptation to environmental stressors. This offers an important starting point for the quantitative analysis of lateral gene transfer in natural settings and its incorporation into evolutionary and ecosystem studies and modeling.

## Introduction

Lateral gene transfer (LGT) is a major evolutionary process in Bacteria and Archaea, with impacts spanning the spread of antibiotic resistance among human pathogens, the emergence of new capabilities in planetary biogeochemical cycles, and serving as a fundamental driver of microbial speciation [1–3]. Recent discoveries extend the repertoire of classical LGT mechanisms—natural transformation, conjugation, and transduction—with a plethora of previously unknown processes and vectors, such as gene transfer agents, phage-inducible chromosomal islands, extracellular vesicles, tycheposons, and likely other, yet undiscovered modes [4–6]. However, quantification of *in situ* rates of LGT has proven challenging. Existing estimates of LGT rates are primarily based on studies of model bacterial cultures grown under laboratory conditions [7, 8], model genetic elements employed in the field [9], or inferences about a limited set of genes in genomes of cultured isolates [10–12]. Due to biases inherent in these approaches, current rate estimates are likely not fully representative of the diversity of microorganisms and evolutionary processes in nature.

Complex microbiomes carry millions of genes that collectively comprise the enormous pangenomes of constituent lineages, and most of these genes are exceedingly rare [13, 14]. Comprehensive tracking of lateral transfers of such rare genes using traditional phylogenetic approaches requires bias-free genome sampling at scales that remain unattainable in contemporary microbiology. Another challenge is the complex and poorly understood microbial biogeography, which exhibits structure from microscopic to global scales and obscures the discrimination of biological (e.g. evolutionary distance) and physical (e.g. cell encounter rate) factors impacting the distribution and exchange of genetic material [15, 16]. Subsequently, even approximate LGT rates and phylogenetic range in natural, complex microbiomes remain largely unknown, representing a major knowledge gap in microbiology [1–3].

Marine planktonic Bacteria and Archaea (prokaryoplankton) constitute two-thirds of the ocean’s biomass and play essential roles in the global carbon cycle, nutrient remineralization, and climate formation [17]. Prior studies have suggested that LGT is important in the evolutionary patterns of specific prokaryoplankton lineages [18–20]. The global mixing of the ocean by thermohaline circulation every 1000–2000 years and surface currents acting at much shorter time scales [21] minimize the impact of dispersal limitations on long-term evolutionary processes of prokaryoplankton. Prokaryoplankton sister cells are estimated to separate by several kilometers each week, on average [20]. Although environmental factors can drive regional differences in the lineages’ relative abundances [22], cells inhabiting epipelagic environments in tropical and subtropical latitudes are efficiently dispersed and differ genetically from cells inhabiting higher latitudes and greater depths, with the physicochemical environment driving these biogeographic patterns [14, 23]. Genomic sequencing of 6236 individual prokaryoplankton cells from a single 0.4 ml seawater sample recovered a substantial fraction of the total coding potential of prokaryoplankton inhabiting this zone [14]. It is likely that prokaryoplankton inhabiting the tropical and subtropical epipelagic have been experiencing a rather stable environment for tens of millions of years [24]. This offers a less complex and more stable system for studies of microbiome-wide evolutionary processes compared to the more compartmentalized and temporary environments, such as soils and host-associated holobionts.

A recently reported collection of 12 715 randomly sampled single amplified genomes (SAGs) from 28 globally distributed samples of tropical and subtropical, epipelagic ocean water, collectively called Global Ocean Reference Genomes—Tropics (GORG-Tropics), offers a unique opportunity for quantitative, microbiome-wide tracking of gene distribution at the resolution of individual cells [14, 25]. No two GORG-Tropics cells were found to contain identical gene content, and extensive LGT was hypothesized as a plausible explanation for this observation. Here, we harness GORG-Tropics and a novel computational approach, which we call Discrepancy in Gene Share (DIGS), to estimate *in situ* rates of net LGT in the epipelagic zone of tropical and subtropical ocean. First, we determined the fraction of highly similar genes in each pair of GORG-Tropics genomes and compared it against data derived from an LGT-free model. Then, we used discrepancies between the observed and modeled data in the context of evolutionary distances of compared genomes to estimate the average net rate of LGT in this microbiome. Our analyses indicated that marine prokaryoplankton acquire and retain ~13% of their genes through LGT every 1 million years, translating to a net rate of ~250 genes L^−1^ day^−1^, with most transfers occurring among closely related cells. Subsequently, we applied established methods to identify examples of LGT in GORG-Tropics. We explored how these events impact DIGS, which revealed LGT of both ecologically relevant “flexible” genes as well as “core” genes that are frequently used as phylogenetic anchors, including the 16S rRNA gene.

## Materials and methods

### Genome data sources

The main dataset utilized in this study is Global Oceans Reference Genomes Tropics (GORG-Tropics), which consists of 12 715 partial genomes of individual cells of bacteria and archaea obtained through a random selection from 28 globally distributed samples of tropical and subtropical, photic ocean water column [14]. In order to reduce spurious results and accommodate CPU-intensive data analyses, we selected for this work 861 GORG-Tropics SAGs that had ≥80% estimated completeness and contained the 16S rRNA gene (Data S1). This GORG-Tropics subset contained all predominant taxonomic groups of phototrophic and heterotrophic prokaryoplankton represented in the complete GORG-Tropics dataset, with a notable overrepresentation of Pelagibacterales and HIMB59 and an underrepresentation of other taxa (Fig. S1). The selected set of SAGs was found to contain no detectable sequence contamination by the original study [14], which was confirmed by a subsequent application of additional quality control algorithms [25]. Open reading frames and their annotations were extracted from previously published gbk files for GORG-Tropics SAGs [14]. Clusters of Orthologous Genes (COG) categories were assigned to open reading frames using the eggNOG-mapper 2.1.2 [26].

### Genome-wide comparisons

Average nucleotide identity (ANI) of genome pairs was calculated using the ANIb method within pyani version 0.2.9 [27] (Data S2). Although computationally more demanding than alternative approaches, this technique offers superior sensitivity across a broader range of ANI values [28]. ANI comparisons with <3% overlap in either direction and self-comparisons were removed. Genome nucleotide difference (NDgenome) was calculated as NDgenome = 1-ANI.

The genome-wide amino acid identity (AAI) and the fraction of orthologous genes were determined with CompareM version 0.0.23 [29] using default parameters. CompareM uses reciprocal best hits to identify orthologous pairs. The genome-wide amino acid difference (AADgenome) was calculated as AADgenome = 1-AAIgenome.

To evaluate the accuracy of NDgenome and AADgenome estimates, we generated a set of mock genomes by *in silico* evolution of pairs of closely related GORG-Tropics genomes (Data S3). We modeled the variation of evolutionary rates across a genome by introducing genomic changes only through point mutations, with no LGT or other genome rearrangements. We detail the simulation procedure below under the “Genome Evolution Simulation” section. We found that the expected and estimated NDgenome values above ~25% NDgenome had major discrepancies, whereas there was a good agreement between the expected and observed AADgenome over the entire 0%–60% range of values (Fig. S2). Subsequently, only AADgenome values were considered in pairwise comparisons of genomes >20% NDgenome.

### Modeling of LGT-free nucleotide substitutions

**Figure 1:**
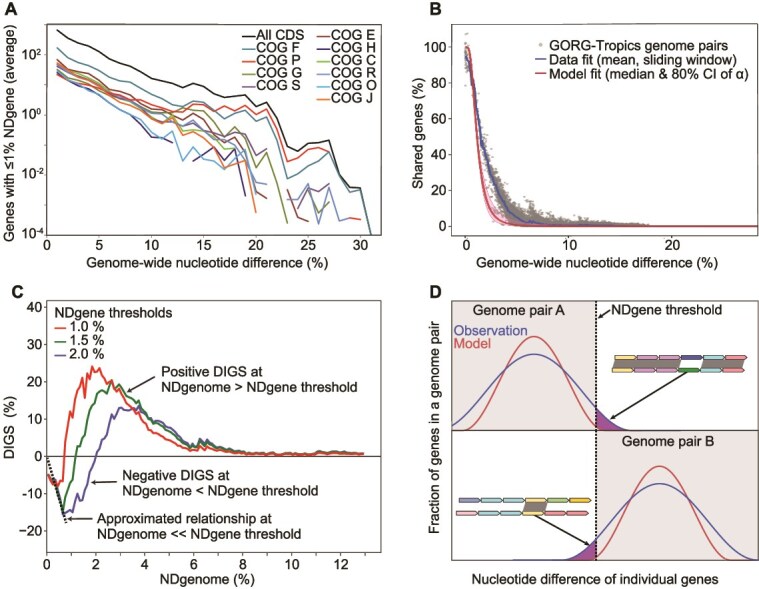
Quantification of LGT rates in GORG-tropics. (A) Relationship between average counts of protein-coding genes with <1% NDgene and NDgenome in pairs of GORG-tropics genomes, estimated at 1% NDgenome increments. Colored datasets correspond to 10 most abundant COGs. (B) Relationship between NDgenome and the observed versus model-predicted fraction of genes with <1% NDgene. The model excludes LGT and gene loss but includes mutation rate heterogeneity (see Fig. S1 for validation of model) and corrects for genome incompleteness (the reasons for >100% observations). The shaded region shows model results with 80% range of the α parameter of gamma distribution. (C) Relationship between NDgenome and DIGS—the discrepancy in gene share between observation and model prediction—estimated for genes with <1%, <1.5% and < 2% NDgene. The dotted line represents a linear approximation of the relationship between NDgenome and DIGS at NDgenome ≪ NDgene threshold, with the intercept set to zero. This line corresponds to a change of DIGS by −26% per 1% NDgenome. (D) Schematic representation of the impact of LGT on DIGS in genome pairs with NDgenome << NDgene threshold (upper plot) and NDgenome > > NDgene threshold (lower plot). The blue and red bell-shaped lines represent the observed and LGT-free-modeled distributions of NDgene. The two white quadrants indicate NDgene regions in which differences between the observed and modeled gene share (DIGS; purple-shaded areas) are detectable. Insets illustrate alignments of genome regions containing laterally transferred genes that contribute to either negative (upper plot) or positive (lower plot) DIGS. In insets, same gene color indicates homology and grey shading indicates regions with NDgene < NDgene threshold.

Let $D$ be the average DNA divergence between homologous genes across two genomes, measured as the proportion of non-identical positions, and note that this quantity approximates NDgenome. We model how $D$ impacts the normalized gene nucleotide difference (NDgene) between two orthologous genes. Following a long tradition [30, 31], we assume that the per-position probability of (observed) substitution for each individual gene is ${r}_i\ D$. Here, ${r}_i$is specific to that gene (i.e. ${r}_i>1$ for a fast-evolving gene) and is distributed according to a Gamma distribution with shape and scale parameters both set to $\alpha$, which results in mean 1 and variance $\frac{1}{\alpha }$. Note that the Gamma distribution is found in previous analyses to be a good fit not only for variation of rates across sites but also the variation of rates across genes [31, 32]. Then, we can assume that the observed number of non-matching positions between the two genes follows a Poisson model with $\lambda ={r}_i\ D\ L$ where $L$ is the gene length. Let the “expected gene shared” (EGS) be the probability of the normalized NDgene between two genes being no more than a threshold $\rho$ (e.g. $\rho =0.01$). Under this model, for small enough $\rho$ (where back-mutation probability can be ignored), for a given average divergence $D$ and rate variation parameter $\alpha$, EGS can be calculated as:


$$ EGS\left(\rho; D,\alpha, L\right)={\int}_0^{\rho L}{\int}_0^{\infty}\frac{(rDL)^x{e}^{-(rDL)}}{x!}{f}_G\left(r;\alpha, \alpha \right)\ drdx $$

where ${f}_G\left(x;\alpha, \alpha \right)$ is the probability density function (pdf) of the Gamma distribution. We calculated this quantity using Mathematica (v.12.1.0.0) (exact scripts available under the github repository) for a range of $D$ values with $\rho =0.01$ (or $\rho =0.015\ or\ \rho =0.02$) to capture near-identical genes (Fig. 1B). Values of $\alpha$ and L were estimated from the data.

For L, to make sure the impact of gene length variation was minimal, we restricted our analyses to genes between 500 bp and 1500 bp, and set L = 904.3, which was the average length of genes in this window.

For $\alpha$, we used variance of gene identity estimates for pairs of genomes. Under the gamma model of rate variation, ${\left(\frac{1-D}{\sigma}\right)}^2$gives a valid estimate of $\alpha$. For each genome pair, we used this equation to estimate $\alpha$ and examined the distribution (Fig. S3A). For each pair of genomes, we calculated $D$ and $\sigma$ by taking the average and standard deviation of 1 minus percent identity, as reported by blastn. We noticed that blastn results include a set of potentially spurious cases where $D$ is computed from only a handful of blast hits (Fig. S3B). In these cases, $D$ and $\sigma$ estimates are unreliable. Thus, we empirically determined a simple criterion: $\# hits>800-5000\ D$ to separate spurious results (Fig. S3B), which filtered 61 155 out of 312 457 genome pairs. Estimates of $\sigma$ are less reliable when there has not been enough time for divergences to be observed (low $D$), or when aligning is difficult due to high levels of divergence (high $D$). Thus, we focused on the distribution of estimated alpha for $0.05<D<0.2$ and at least five blastn hits. We used 10%, 50%, and 90% quantiles (i.e. the 80% CI) of the $\alpha$ distribution in our analyses, which were 3.31, 5.28, and 7.77 (Fig. S3A).

### Calculating Discrepancy in Gene Share

To quantify the level of LGT, we define a “Discrepancy in Gene Share” (DIGS) metric calculated as the difference between the modeled and observed fraction of shared genes above a specified NDgene per NDgenome level. We compared the results of the model $EGS\left(\rho; D,\alpha, L\right) $ to the values computed from real or simulated genomes. Let “observed genes shared” (OGS) between genomes A and B at a NDgene threshold $\rho $ be

OGS(A,B; $\rho$) = the portion of genes from A that are found in B with NDgene below $\rho$.

We performed the search for highly similar genes using reciprocal BLASTn version 2.9.0+ [33] between the open reading frames of genome pairs. BLAST hits were maintained if percent identity was equal or greater than the set percent identity threshold $\rho$ across an alignment length that was greater than or equal to 80% of the length of either A or B. Then, for a small range of NDgenome $\left(D-\varepsilon, D\right]$ (default $\varepsilon$ =0.1%), let $GP(D)$ be the set of genome pairs with NDgenome in that range:


$$ \textrm{DIGS}\left(\textrm{D},\rho \right)=\frac{1}{\mid GP(D)\mid}\sum_{A,B\in GP(D)}\textrm{OGS}\left(\textrm{A},\textrm{B};\rho \right)-\textrm{EGS}\left(\rho; D,\alpha, L\right) $$

As noted earlier, only genes with length between 500 and 1500 bp were considered in $GP$ and OGS and parameters of the model ($\alpha, L$) were calculated accordingly as described earlier.

Negative DIGS values indicate fewer near-identical genes than modeled, reflecting a net-negative impact of LGT on gene sharing; positive DIGS values, by contrast, indicate more near-identical genes than modeled/expected. The model was implemented with NDgene thresholds of $\rho$ = 1%, 1.5%, and 2%. The range of NDgene thresholds we chose for this study is based on practical constraints. Lowering the NDgene threshold is desirable, enabling LGT detection among closer relatives and at finer evolutionary time intervals. However, GORG-Tropics does not contain sufficient genome pairs with NDgenome <1% to produce meaningful results with NDgene below this threshold.

An additional complication is the incompleteness of the GORG-Tropics genome assemblies. To account for the fact that our genome assemblies were not complete, we used an estimate of genome completeness obtained using CheckM [34]. Whereas CheckM uses single-copy genes to estimate completeness, we have no reason to believe that these numbers are systematically under- or overestimated compared to the rest of the genome (Fig. S4). Thus, we assumed these ratios apply to every gene and evaluated the impact of completeness in simulations (Fig. S5). Let ${n}_A$ and ${n}_B$ be the number of observed genes in two genomes A and B. Let ${c}_A$ and ${c}_B$ be their estimated completeness levels. Let the observed number of shared genes with divergence below $\rho$ according to blastn be $s$. To compute the percentage of genes that would be shared in the full genome, we use $OGS\left(A,B;\rho \right)=\frac{s}{2}\left(\frac{1}{n_A{c}_A}+\frac{1}{n_B{c}_B}\right)$. This equation is obtained using the following calculations and assumptions. Let

-
$s=$ number of observed shared genes
-
${s}_A=$ number of shared genes observed in A but missing from B; ditto for ${s}_B$
-
${s}^{\prime }=$ number of shared genes missing from both A and B
-
${u}_A=$ number of genes observed in A but not shared with B; ditto for ${u}_B$
-
$u{\prime}_A=$ number of genes missing in A and not shared with B; ditto for $u{\prime}_B$

Note that

-
${n}_A=s+{s}_A+{u}_A$ ; ditto for ${n}_B$
-
${N}_A=s+{s}_A+{s}_B+{s}^{\prime }+{u}_A+{u}_A^{\prime }$ = total number of genes in (complete) A; ditto for ${N}_B$
-
${c}_A=\frac{n_A}{N_A}$ ; and thus ${N}_A=\frac{n_A}{c_A};$ditto for ${c}_B$

We define the true percentage of shared genes if we had complete genomes as


$$ shared=\left(\frac{s+{s}_A+{s}_B+{s}^{\prime }}{N_A}+\frac{s+{s}_A+{s}_B+{s}^{\prime }}{N_B}\right)\Big/2 $$

Assuming completeness is random with respect to whether genes are shared, and the impact of genome incompleteness is the same among shared and unshared genes, we can write

-
${c}_A=\frac{s+{s}_A}{\left(s+{s}_A+{s}_B+{\textrm{s}}^{\prime}\right)}$ and thus, $s+{s}_A+{s}_B+{\textrm{s}}^{\prime }=\frac{s+{s}_A}{c_A}$.

-
${c}_A=\frac{s}{\left(s+{s}_A\right)}$ and thus, $\left(s+{s}_A\right)=\frac{s}{c_A}$.

Replacing these in the equation with shared genes gives


\begin{align*} shared&=\frac{1}{2}\left(\frac{s+{s}_A+{s}_B+{s}^{\prime }}{N_A}+\frac{s+{s}_A+{s}_B+{s}^{\prime }}{N_B}\right)\\&=\frac{1}{2}\left(\frac{s+{s}_A}{n_A}+\frac{s+{s}_B}{n_B}\right)=\frac{s}{2}\left(\frac{1}{n_A{c}_A}+\frac{1}{n_B{c}_B}\right). \end{align*}

### Genome evolution simulations

We performed two types of simulations to examine the accuracy of a) NDgenome and AADgenome calculations and b) our LGT-free nucleotide diversity model. We perform these simulations with similar procedures, with some differences mentioned below.

*a) Examination of NDgenome/AADgenome methods.* For a pair of closely related genomes X and Y, choosing one (say, X) arbitrarily as the base, we added mutations to it in the amino acid space and back-translated them to nucleotides to get X’. We then computed both the NDgenome and AADgenome distance from X’ to Y and compared it to the ground truth, which is defined as the actual number of mutations added during the simulation, divided by the length. We computed ground truth for both nucleotide and AA sequences.

To add mutations to X, we first draw a relative rate multiplier ${r}_i$ for each of its genes from a gamma distribution (with an $\alpha$parameter; as before). Given an AADgenome level $\mu$ and total genome length L, we randomly selected $n=\mu\ L$ amino acids (sampling without replacement) to mutate using BLOSUM62 model; each amino acid is selected with a probability determined by the rate ${r}_i$ of the gene it belongs to. When placing mutations, we avoided adding or removing both start and stop codons to keep gene boundaries intact. We also avoided interrupting the reading frame: when encountering a pair of genes that overlap each other (possibly with different reading frames), we did not mutate the overlapping region. We did not mutate intergenic regions (~5% of the genome, on average). After making all the mutations in the amino acid space, we back-translated the sequences to DNA. If there was a codon identical to the original genome (i.e. for positions that were not mutated), we chose it; otherwise, we randomly selected a codon with weight proportional to 4-d, where d is its Hamming distance to the codon present in the original genome. This back-translation gave us the nucleotide genome, which we used to define and compute the ground truth for NDgenome.

We selected five phylogenetically diverse pairs of closely related SAGs (Data S3) and added mutations at levels ranging from $\mu =0.01$ to $\mu =0.69$ with a step size of 0.02 and $\alpha =22$ to the first genome. We computed $\alpha =22$ based on AADgenome of real genomes computed using compareM; specifically, we computed an $\alpha$ for each genome using $\left( \frac{1 - \bar{d}}{\bar{\sigma}} \right)^2$here $\underset{\_}{\sigma }$ and $\underset{\_}{d}$are the mean and standard deviations of amino acid identity of the gene as computed by compareM. Note that the $\alpha$ used here is higher than $\alpha$ used for nucleotide data due to less variance in rates of amino acid evolution.

*b) Examination of the LGT-free nucleotide diversity model.* The simulations used to test the LGT-free model differed from the *NDgenome/AADgenome* simulations in several ways. Given our model is for nucleotide evolution, rather than applying mutations in amino acid space, we mutated the genome in nucleotide space. However, we tracked codons and ensured that stop and start codons did not mutate. The mutations followed a simple Jukes-Cantor model (i.e. are fully symmetric). Here, instead of back-translating, we translated the mutated DNA to AA. Because simulations were in nucleotide space, we set $\alpha$ = 5 to match the estimate from the real data. Unlike previous simulations, overlapping areas between genes were mutated, but only if they did not alter the start and stop codons. The rate multipliers of these overlapping areas are randomly selected among the genes that include them.

In these simulations, instead of starting from two genomes X and Y, we started from one genome, X (Data S4), and mutated it at various nucleotide diversity levels (0%, 0.05%, 0.1%, 0.2%, … 1%, 2%, 3%, …19%) to obtain X_0,_ X_1_, X_2, …_ X_30_. We then compared all pairs of X_i_, X_j_ genomes using the same blastn pipeline described earlier to compare real genomes. In these simulations, we ensured that each gene in the base genome X was assigned a fixed rate, which was used for all X_i_s. In these simulations, we also modeled the impact of genome incompleteness. To do so, for each genome, we created five incomplete versions by randomly selecting an incompleteness level (p) from the distribution of incompleteness of real genomes (mean = 12%). Instead of removing one chunk from the genome, we drew the number of removed chunks n from Poisson (mean = 17), the length of each region from Poisson (mean = p*L/n) where L is the total length of the base genome. The starting position of each chunk was selected uniformly from the set of all possible starting positions. We estimated completeness using CheckM for these genomes and divided this value by the CheckM estimate of completeness of the original genome (before removal) to obtain the $1-\textrm{c}\ (\textrm{the}\ \textrm{in}\textrm{completness}$ parameter mentioned earlier). We show results with and without the added incompleteness. Finally, instead of starting from five base genomes, we began with a set of 28 genomes (Data S4), selected to have high levels of completion, to obtain sufficient points for examining the model fit. Once all 30 child genomes were simulated, we compared them using the same pipeline used to analyze the real data and examined the model fit. Also, note that $\alpha$ was estimated for simulated data using the same procedure used on real data (instead of using the known $\alpha$ value), but the estimate (5.48) was close to the true value (5).

### Phylogenetic analyses of the *phnCDEubiE* operon

The 3263 nucleotides of the *phnCDEubiE* operon of HIMB59 SAG AG-893-E15 were used as a query in a BLASTn search for matches in GORG-Tropics and in NCBI nt databases, producing 20 and 0 contiguous matches of at least 3000 bp in length. These sequences were aligned with MUSCLE [35], and a phylogenetic tree was constructed using IQ-TREE version 2.1.1 under the GTR + G model with approximate Bayes support values (−abayes), which are between 1/3 (indicating no support) to 1, indicating full support among local rearrangements [36].

### Phylogenetic analyses of conserved genes

Protein-coding genes from all SAGs in GORG-Tropics were grouped by Prokka-annotated gene name [37]. The number of SAGs containing genes from each group, average gene length, and average number of copies of each gene group per genome were calculated. We selected 204 protein-coding gene families that were found in the largest number of SAGs and had less than or equal to 1.25 mean copies per genome.

For each gene family, we performed multiple sequence alignment using UPP version 4.5.1 [38], then removed sites with >95% gaps and sequences with >66% gaps. Next, we inferred a preliminary gene tree for each gene family using FastTree version 2.1.10 [38]. Next, we removed outlier sequences using TreeShrink version 1.3.9 [39], then repeated the alignment step to get high-quality sequence alignments. Next, we inferred the final gene trees using IQTree version 1.6.12 [40]. Finally, we used ASTRAL-Pro [41] to estimate a putative species tree. The pipeline used here was similar (but not identical) to other studies of bacterial genomes [42]. Multiple sequence alignment of the 16S rRNA genes was performed using SINA version 1.2.11 [43] and followed by alignment trimming with trimAl version 1.4rev15 [44]. Maximum likelihood trees were inferred using RAxML version 8.2.10 [45] with default settings and 1000 bootstrap replicates.

*Discordance with AADgenome.* To examine LGT across all conserved genes, we contrasted AADgenome and gene trees. We used AADgenome between pairs of 861 genomes with estimated >80% genome completion by CheckM [34], denoted here by $D\left(A,B\right)$ for two genomes A and B. Also, let ${d}_i\left(a,b\right)$ denote the total branch length between leaves a and b on the gene tree $i$. For each query genome $Q$, we found the closest species to $Q$ according to AADgenome among all the remaining genomes and called it $M$. We found the three closest leaves on each gene tree $i$ to $Q$, according to gene tree path lengths, denoting corresponding genomes by ${N}_{1,i},{N}_{2,i},{N}_{3,i}$. We note that $D\left(Q,{N}_{j,i}\right)\ge D\left(Q,M\right)$ for $1\le j\le 3$ by definition. If there is no LGT and gene trees match the species tree, we expect $\varDelta \left(Q,{N}_j\right)=D\left(Q,{N}_j\right)-D\left(Q,M\right)$ values to be small, especially when gene tree distances (${d}_i\left(Q,{N}_j\right)$) are also low. When LGT moves a gene to an evolutionarily distant genome, $D\left(Q,M\right)\ll D\left(Q,{N}_j\right)$, despite the closeness on the gene trees, leading to high $\varDelta$ values. Thus, we depicted $D\left(Q,M\right)$ versus $D\left(Q,{N}_j\right)$ for $1\le j\le 3$ and showed ${d}_i\left(Q,{N}_j\right)$ as color. Deviations from the unity line are evidence of LGT, especially when ${d}_i\left(Q,{N}_j\right)$ also points to a low value, with $\varDelta$ corresponding to the vertical distance from the unity line. When there were multiple copies of $Q$, we repeated this process for each copy independently.

We designed a statistical test to ask if divergences between gene trees and AADgenome are statistically significant. We empirically detected that the distribution of $\varDelta$ values were close to that of Exponential plus some outliers (Fig. S6). Using the Hellinger metric of divergence, we confirmed that the empirical distribution of $\varDelta$ was less divergent from the Exponential distribution compared to both Log-Normal and Gamma distributions (two other common distributions on positive values). To fit an exponential distribution to the data, we only need one parameter, which is often set to the inverse of the mean. However, because the mean is sensitive to outliers, and to make the estimate robust to the outliers, we used median and set $\hat{\lambda}=\frac{lnln\ 2\ }{median\left(\varDelta \right)}$, which is a valid and more robust estimator. For each gene tree, we computed a separate exponential distribution and assigned a P-value to each observed $\varDelta$ value using the cumulative distribution function of the Exponential distribution: $p={F}_{exp}\left(\delta; \hat{\lambda}\right)$. Because we performed many statistical tests, to avoid high levels of false positives, we corrected for multiple hypothesis testing using the False Discovery Rate (Benjamini-Hochberg) approach across all the genes and reported the resulting P-values.

*Tree discordance.* To compare the discordance of the 16S gene tree with the species tree versus AADgenome, we first created 3000 genome subsets, each containing 10 SAGs such that each subset has a controlled range of pairwise AADgenome. To do so, we used the single-linkage agglomerative clustering algorithm implemented in TreeN93 github commit number a4e2bfc8a0bd573d484165b7e99a53aa5eb443b9 [46] to build a hierarchy of the genomes. At any threshold of AADgenome, all genomes within that threshold belong to a subtree of the single-linkage tree. We cut this tree at different thresholds of AADgenome and chose 10 individual genomes at random from different subtrees. This allowed us to control the pairwise AADgenome of the 10 genomes within a certain range. By varying the threshold between 0.5% and 29.5% (with each step equal to 0.5%), we obtained 60 groups of subtrees and randomly sampled 50 subsets of size 10 from each group. This procedure produced 3000 subsets that covered the range between 0.01% and 55.2% mean pairwise AADgenome (average: 26.2%). For each of the 3000 subsets, we pruned the original 16S tree, its bootstrap replicates, and the ASTRAL-pro species tree to include only the 10 selected SAGs. We then used tqDist version 1.0.2 [47] to compute quartet matches. We computed the quartet distance between the restricted 16S rRNA gene ML tree and the restricted ASTRAL-pro species tree (${q}_s$) and the average quartet distance between the restricted 16S rRNA gene tree and each of its bootstrap replicates (${q}_{\varnothing }$). The discordance captured by ${q}_{\varnothing }$ is due to uncertainty in gene tree estimation and compounds the discordance observed in ${q}_s$. To remove the impact of such noise, we reported ${q}_s-{q}_{\varnothing }$.

### Data and materials availability

The primary dataset, GORG-Tropics, is available at the European Nucleotide project PRJEB33281 and the Open Science Framework project pcwj9. The code used to generate data and figures (including gene trees and the species tree) is available at https://github.com/smirarab/GORG-LGT, https://github.com/BigelowLab/GORG-HGT-bioinfo

## Results

### A model-based approach for quantifying LGT rate in natural populations

To determine how gene conservation patterns might appear in the absence of LGT, we modeled the expected gene conservation in pairs of GORG-Tropics genomes over a range of genome-wide nucleotide differences (NDgenome; inverse to average nucleotide identity, ANI) while accounting for rate variation using a standard Gamma distribution with the shape parameter α (i.e. the inverse of the variance of one-centered substitution rate multipliers across genes). This parameter was estimated using pair-wise BLASTn [33] analyses of GORG-Tropics genomes (see Materials and Methods). Our substitution-only model accounted for differences between NDgenome and the fraction of differing nucleotides of individual genes (NDgene) due to stochastic substitutions with heterogenous rates, as well as for genome incompleteness. The model predicted the fraction of genes expected to be shared below a given NDgene level, considering overall pairwise differences in NDgenome. LGT and gene loss were excluded from the model, and we expected these processes to lead to measurable discrepancies between the modeled and observed trends.

To examine LGT rates, we compared our substitution-only model to the fraction of shared genes and NDgenome calculated for 861 high-quality prokaryoplankton genomes from the GORG-Tropics collection. We did so by using the DIGS measure described earlier. Both the real data and the substitution-only model indicated a decline in the fraction of near-identical genes in genome pairs with increasing NDgenome (Fig. 1A-B). However, substantial discrepancies in gene share were observed between the modeled and the observed values in GORG-Tropics (Fig. 1C). To ensure that these discrepancies are not an artifact of computational tools, we applied the same algorithms to genomes evolved *in silico* without LGT. This resulted in a NDgenome-to-shared-gene relationship that was in good agreement with the LGT-free probabilistic model whether we simulated complete or incomplete genomes, indicating that our DIGS estimates are robust and reflect true LGT impacts on GORG-Tropics (Fig. S5). Furthermore, to confirm that the workflow used to generate GORG-Tropics does not introduce biases arising from uneven recovery of various genome regions, we examined SAGs of a monoclonal culture of *Prochlorococcus marinus* and found that the employed methodology does not materially impact gene share estimates (Fig. S4).

We next explored the implications of these findings at a selected gene NDgene threshold of 1%. When genomes A and B have NDgenome ≪1%, the model predicts most genes to have NDgene <1%. A lateral acquisition of genes by cell line A with NDgene >1% relative to B and vice versa since their last common ancestor reduces the fraction of shared genes with NDgene <1%, thus contributing to negative DIGS values (Fig. 1D). Meanwhile, lateral acquisition of genes by cell line A with NDgene <1% relative to B and vice versa does not change the fraction of genes with NDgene below the set threshold and, therefore, has no impact on DIGS. Thus, a decrease of DIGS with increasing NDgenome in the NDgenome range ≪ NDgene threshold corresponds to the continuous accumulation of laterally acquired genes through time either through non-homologous recombination (acquisition of new genes) or homologous gene replacements sourced from organisms with NDgenome predominantly above the set NDgene threshold. Gene decay and loss also contribute to DIGS reduction. In our study, the application of NDgene thresholds 1%, 1.5%, and 2% produced similar regression coefficients in the DIGS versus NDgenome relationship at each NDgenome ≪ NDgene threshold, averaging at ~26% per 1% NDgenome (dotted line in Fig. 1C). Considering that average genome sizes are rather stable over relatively short evolutionary time scales [10], we can then assume that rates of gene acquisition and loss are approximately equal, suggesting that ~50% of the observed DIGS decrease is due to lateral gene acquisitions. This is likely an over-correction, as homologous gene replacements sourced from distant donors do not require a balancing gene loss to reduce DIGS values. Another scenario that may lead to DIGS underestimates is when the same gene is transferred from a donor C independently to two recipients, A and B, in the time equivalent < NDgene threshold. However, given the low NDgene thresholds used in our study, such parallel transfers are expected to be rare and not have a major impact on DIGS estimates. An important consideration is that DIGS is a result of gene gain and loss in both cell lines A and B, with ~50% of the observed gene content differences coming from each. This implies that, on average, marine prokaryoplankton genes that transfer laterally and *are retained* (i.e. the net rate of LGT) in a cell line over a period of time that takes to accumulate 1% NDgenome is ~26% × 0.5 × 0.5 = 6.5% of their gene content.

Our DIGS-based analysis provides microbiome-wide estimates of gene acquisition rates. Assuming that the average amino acid substitution rate for bacteria, ~2% Ma^−1^ [48, 49], is similar in marine prokaryoplankton, given the average per-genome gene count of 1377 [14], and given the similarity of amino acid difference (AADgenome; inverse to amino acid identity, AAI) and NDgenome values at their low range (Fig. S3), we can translate the LGT rate of 6.5% genes per 1% NDgenome (see above) to ~180 genes (13%) being acquired and retained by a cell line per million years (1377 × 0.065 × 2 = 179). Considering the generation length of an average prokaryoplankton cell of ~10 days [50], this corresponds to ~5 × 10^−6^ genes acquired laterally and retained per cell and generation (179 × 10 / [10^6^ × 365] = 4.9 × 10^−6^). Given ~5 × 10^8^ L^−1^ prokaryoplankton cells in the surface ocean [51] and assuming that the average impact of laterally acquired and retained genes is near-neutral [1–3], we make a rough estimate of the average rate of net LGT at ~250 genes L^−1^ day ^−1^ in the tropical and subtropical, epipelagic ocean (179 × 5 × 10^8^ × 10^−6^/ 365 = 245).

When the NDgenome of genomes A and B is substantially above the set NDgene threshold, the NDgene of most homologs is expected to exceed this threshold in the absence of LGT (Fig. 1D). Our model indicates that lateral acquisitions of genes by A with NDgene above the threshold of 1% relative to B, and vice versa, have no impact on DIGS. Instead, DIGS is affected positively when genes are acquired laterally by recent ancestors of A from close relatives of B with NDgenome <1% relative to B and vice versa. After a transfer, the laterally acquired genes continue accumulating mutations in the recipient’s cell line and eventually exceed the NDgene threshold, thus blurring the impact of older LGT events on DIGS. Consequently, recent LGT events (to A from donors similar to B or vice versa) dominate the contribution to positive DIGS values. We observed a negative relationship between DIGS and NDgenome at the NDgenome ≫ NDgene threshold (Fig. 1C), indicating that LGT rates decline with the evolutionary distance between the donor and the recipient, in agreement with prior laboratory-based findings [7, 8]. However, positive DIGS values, indicative of recent LGT, extended well beyond 5% NDgenome, and no discontinuities in either DIGS or raw counts of near-identical genes (Fig. 1A) were observed at the 5% NDgenome level currently used as a nominal definition of bacterial species [52].

### LGT of both “core” and “flexible” genes within marine prokaryoplankton

A key advantage of DIGS is that it provides a framework for examining LGT rates independent of traditional phylogenetic approaches, which currently cannot be applied to the exceedingly rare genes that dominate prokaryoplankton pangenomes [13, 14]. However, this method is limited to providing only the overall LGT rate and is not designed to determine whether a specific gene in a particular genome has or has not been subject to recent LGT. Therefore, we used previously established approaches to identify diverse examples of LGT in flexible and core genes in GORG-Tropics and use them to illustrate patterns influencing DIGS values.

**Figure 2:**
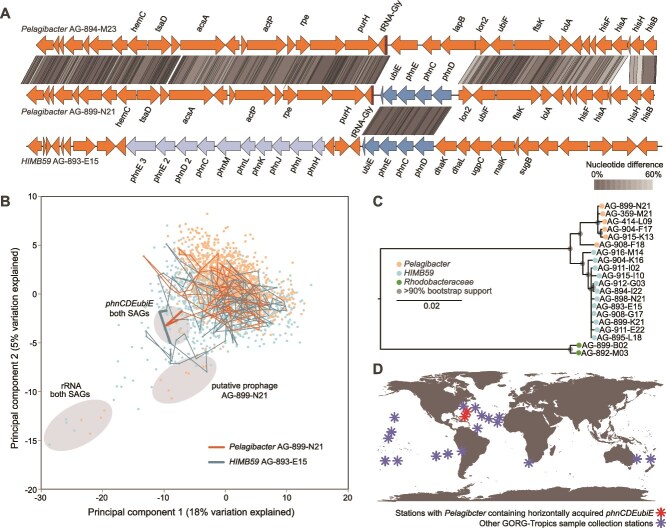
Evidence for recent LGT and non-homologous recombination of an organophosphate transporter operon *phnCDEubiE* between HIMB59 and *Pelagibacter*. (A) Presence of *phnCDEubiE* genes in only one of the two *Pelagibacter* genomes AG-894-M23 and AG-899-N21 with 5.3% NDgenome; and the share of these genes with 1.6% NDgene in *Pelagibacter* AG-899-N21 and HIMB59 AG-893-E15 genomes with 29.4% NDgenome. The *phnCDEubiE* operon that is shared by HIMB59 and *Pelagibacter* and the additional phn operon that is present only in HIMB59 are colored in dark and light blue. (B) Principal components 1 and 2 of tetramer frequency in *Pelagibacter* and HIMB59 SAGs with a shared *phnCDEubiE* operon. Each data point represents a 2 kbp window analyzed at 1 kbp step. Thick lines connect windows containing *phnCDEubiE* operons and thin lines connect other windows on the same contigs. Grey background indicates notable outlier regions. (C) Maximum likelihood tree of the *phnCDEubiE* operon. Included are all genome regions in the analyzed GORG-tropics SAGs that lack <100 bases relative to *phnCDEubiE* of AG-893-E15. The homologous region in Rhodobacteraceae was used as an outgroup. The *ubiE* homologs in Rhodobacteraceae SAGs were annotated as “hypothetical proteins” by Prokka. (D) Geographic locations of field samples from which cells containing *phnCDEubiE* were recovered, in the context of all GORG-tropics field stations.

To find examples of recent LGT events with high confidence, we searched for highly similar genes (<2% NDgene) in otherwise evolutionarily distant cells (separate taxonomic Orders). This approach is frequently used in LGT research [11, 12]. Assuming an average amino acid substitution rate for bacteria of ~2% Ma^−1^ [48, 49] and the similarity of AADgenome and NDgenome at their low range (Fig. S7), this analysis captured LGT events that occurred approximately in the past one million years. The only genes found to satisfy our criteria were all part of the phosphonate acquisition operon *phnCDEubiE* shared across taxonomic orders Pelagibacterales and HIMB59 (formerly, HIMB59 was also called AEGEAN-169 or SAR11 group V) (Data S5). Figure 2A illustrates the share of this operon between otherwise diverged genomes AG-899-N21 (*Pelagibacter*) and AG-893-E15 (HIMB59) (29% NDgenome), thus contributing to positive DIGS at high NDgenome. Meanwhile, the same LGT event contributes to negative DIGS in the pair of highly similar (5% NDgenome) genomes AG-894-M23 and AG-899-N21 (both *Pelagibacter*), where one genome lacks this operon.

The *phnCDEubiE* formed a nucleotide tetramer compositional outlier in Pelagibacterales but not in HIMB59, suggesting that this operon may have been transferred from HIMB59 to Pelagibacterales (Fig. 2B). Additional support for the HIMB59 to Pelagibacterales direction of this LGT was provided by: a) the presence of tyrosine recombinases and tRNA genes, which often serve as recombination sites, adjacent to *phnCDEUbiE* in Pelagibacterales but not HIMB59; and b) *phnCDEUbiE* being located close to the C-P lyase complex and another, larger *phn* operon in HIMB59, but not Pelagibacterales. However, a phylogenetic analysis of available *phnCDEubiE* sequences indicated a consistent separation of Pelagibacterales and HIMB59, whereas no other genomes containing closely related *phnCDEUbiE* operon were found in either GORG-Tropics or GenBank (Fig. 2C). This suggests the possibility that the original donor lineage of *phnCDEUbiE* may not have been captured by either GORG-Tropics or other, current genome collections, or it no longer contains this operon, which highlights the limitations of LGT detection using phylogenetic approaches.

Recently, the phosphonate transporter encoded by *phnCDE* received considerable attention for its importance in prokaryoplankton growth in phosphorus-deplete ocean locations [53]. All GORG-Tropics cells that contain *phnCDEUbiE* originate from the Sargasso Sea (Fig. 2D), a particularly phosphorus-limited geographic region [53]. This corroborates an earlier finding of Sargasso Sea *Pelagibacter* being enriched in genes related to phosphorus starvation [22]. It also underscores the importance of a relatively recent LGT of accessory genes in a biogeochemically significant adaptation of ubiquitous prokaryoplankton lineages to a current environmental stressor.

**Figure 3:**
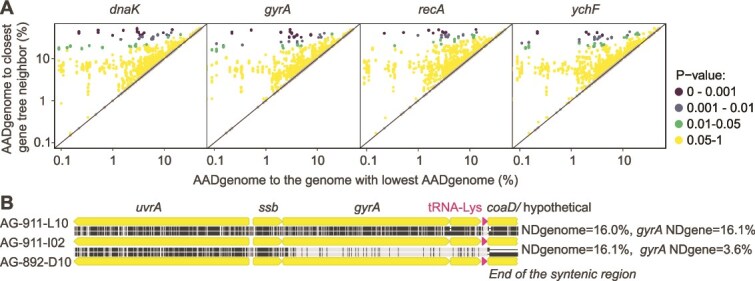
Examples of LGT and homologous recombination of core genes. (A) Discrepancies in AADgenome in pairwise comparisons of genomes with the lowest overall AADgenome in GORG-tropics (x-axis) versus the three closest neighbors on the gene tree (y-axis). The P-values indicate statistically significant deviation from 1:1 line by a parametric test. Displayed are four genes out of 204 on which this analysis was performed. The remaining results can be viewed in Fig. S8. (B) Alignments of genome regions near *gyrA*. Black lines in alignments indicate nucleotide differences. A *gyrA* gene with NDgene = 3.6% was found in *Pelagibacter* AG-911-I02 and AG-892-D10 genomes with 16.1% NDgenome. This gene has much greater NDgene in other pairs of GORG-tropics genomes with similar NDgenome, such as AG-911-I02 and AG-911-L10, indicated in the fig. A recent LGT between ancestors of AG-911-I02 and AG-892-D10 is the most plausible explanation for the low NDgene of *gyrA* in these two genomes. Further evidence is provided by the break of genome synteny near *gyrA* of AG-892-D10, relative to the other two genomes.

The “core” genes, present in all or most members of a microbial lineage, are often considered evolutionarily conserved and therefore used as phylogenomic markers [54]. However, such genes may also involve frequent LGT and subsequent homologous recombination [55]. In search for LGT of core genes in marine prokaryoplankton, we selected 204 protein-coding genes that were found in the largest number of GORG-Tropics SAGs and had only one or very few copies per genome (mean: 1.04) and inferred a gene tree for each of them. We then examined AADgenome between each possible “query” genome and its closest three neighbors on the gene tree. Without LGT, one would expect query neighbors in the gene tree to have the lowest possible AADgenome to the query. Thus, large deviations between AADgenomes calculated between a query and its closest gene tree neighbor versus the query and the genome with the lowest overall AADgenome relative to that query in the dataset indicate LGT. This analysis provided strong statistical support (parametric test, *P* < .001) for the involvement in LGT by at least 189 of the 204 analyzed, conserved genes (Figs. 3A, S8). Figure 3B illustrates such cases with *gyrA,* which encodes DNA gyrase subunit A, an essential bacterial protein that plays a key role in DNA replication and is often used as a conservative marker in phylogenomics [54]. In this case, LGT and homologous recombination of a core gene increase the fraction of highly similar genes (3.6% NDgene), thus elevating DIGS in an otherwise diverged genome pair AG-911-I02 and AG-892-D10 (16% NDgenome).

**Figure 4:**
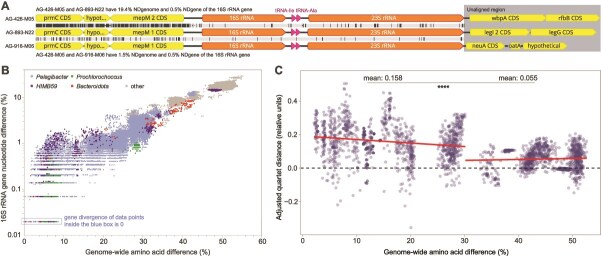
Evidence for LGT of the 16S rRNA gene. (A) Examples of genomic neighborhoods of 16S rRNA genes with 0.5% NDgene in *Pelagibacter* SAGs with NDgenomes equal to 19.4% and 1.5%. (B) Correlation between AADgenome and NDgene of the 16S rRNA gene. Displayed are all GORG-tropics SAG pairs for which AADgenome values could be calculated. (C) Quartet distance between 16S rRNA gene tree and the ASTRAL-pro [59] genome-wide tree. Each dot represents a sample of 10 genomes selected to have similar pairwise AADgenome and the y-axis shows the noise-corrected quartet distance for the subtree restricted to those taxa versus the genome-wide tree. To account for noise in gene trees, we subtract the quartet distance between bootstrap replicates of the 16S rRNA gene tree, which are solely due to uncertainties in gene tree estimation, from quartet distance of the 16S rRNA gene tree versus genome-wise tree. Positive values indicate discordance beyond gene tree estimation noise. Regression models and points of inflection were estimated by change point regression [60] using a stegmented model. Delta quartet distances (mean: ​​15.8%) were significantly greater than 0 at AADgenome <30.2% (*P* < e-15 according to a t-test) and were substantially reduced at AADgenome >30.2% (mean: 5.5%; *P* < e-15).

We asked whether the 16S rRNA genes encoded by GORG-Tropics genomes also carry signatures of LGT. The rRNA operon is generally considered one of the most stable and slowest-evolving genome elements and, therefore, has served as the foundation for microbial genealogy inferences since the 1970s [56]. This assumption was challenged by recent findings of LGT of rRNA genes among closely related strains [57]. Several lines of evidence from the GORG-Tropics dataset suggest that rRNA genes undergo frequent LGT among close relatives in marine prokaryoplankton: (i) inconsistent genomic neighborhoods of near-identical rRNA operons (Fig. 4A); (ii) limited correlation between AADgenome and 16S rRNA gene NDgene at AADgenome below ~25%, including completely identical 16S rRNA genes in cell pairs with up to 9% AADgenome and 14% NDgenome (Fig. 4B; Data S2); and (iii) more than stochastic levels of discordance of 16S rRNA gene phylogeny to a genome-wide tree, especially at low AADgenome values (Fig. 4C). These findings indicate that LGT of the rRNA operon should be taken into account in the SSU rRNA gene-based surveys of microbial composition and in models of microbial evolution.

## Discussion

The application of DIGS on GORG-Tropics indicated that ~13% of prokaryoplankton genes are acquired laterally and retained by their new host every million years, on average. This translates to a net LGT rate of ~250 genes L^−1^ seawater day^−1^. An earlier marine prokaryoplankton LGT study found four orders of magnitude higher LGT rates, but it analyzed gross rather than net rates, which are expected to be much higher, and used model gene transfer agents, donors, and recipients, which were not designed to reflect the full diversity of LGT processes in nature [9]. Several recent reports have examined global LGT patterns in the human microbiome and obtained evidence for detectable LGT occurring within a single human generation [11, 12]. The inter-taxa variability in nucleotide substitution rates [48, 49] and Gamma distribution [31, 32] of natural microbial populations remain poorly constrained and may impact the precision of DIGS-based LGT rate estimates, considering the slight taxonomic biases in the genome completeness-based selection of SAGs for DIGS estimates (Fig. S1). However, this study offers an important starting point for the quantitative analyses of LGT in natural settings and their incorporation into ecosystem studies and modeling.

We found that the frequency of LGT is highest among closely related prokaryoplankton cells, consistent with laboratory experiments using model organisms [7, 8] and studies of the human microbiome [11, 12]. However, no pronounced discontinuities in LGT rates were observed along the NDgenome range (Fig. 1), and ecologically significant gene transfer events were detected across order-level taxonomic distances (Fig. 2). This provides no evidence for discrete, biological species-like units with closed pangenomes in marine prokaryoplankton. The fluidity of prokaryoplankton genomes has important implications for interpreting data produced by contemporary research methods. For example, extensive LGT and lack of pronounced LGT boundaries help explain difficulties in recovering abundant prokaryoplankton taxa in metagenome-assembled genomes [25]. Furthermore, the observed LGT of the 16S rRNA gene and other taxonomic markers suggests caution when interpreting high-resolution microbial diversity patterns based on individual genes.

Considering the generation length of an average prokaryoplankton cell is ~10 days [50], an LGT recipient has the theoretical potential to produce ~2 × 10^28^ progeny—equivalent to the total count of prokaryoplankton cells in the epipelagic tropical and subtropical ocean [51]—in under three years. With sister cells physically separating at a rate of several kilometers each week [20], it is plausible for LGT recipients with large fitness advantages to impact prokaryoplankton communities across globally significant ocean regions on time scales spanning a few years to decades. These time scales are comparable to the global proliferation of laterally acquired resistance genes among human pathogens in response to the introduction of new antibiotics for clinical and agricultural use [3].

The extensive LGT revealed in this study and the large prokaryoplankton pangenomes reported previously [13, 14] imply that, despite the small sizes of their individual genomes, marine microorganisms access vast genetic resources in their adaptation. This underscores the importance of incorporating quantitative analyses of LGT in future studies and modeling of microbially driven processes in the ocean and their responses to natural and anthropogenic stressors, such as global warming, acidification, toxic pollutants, and changes in nutrient availability [58]. The DIGS approach may find future use in the LGT quantification in other microbiomes, such as soils and organismal symbionts, after accounting for the dispersal patterns in such more compartmentalized environments.

## Supplementary Material

Fig_S1_wraf159

Fig_S2_wraf159

Fig_S3_wraf159

Fig_S4_wraf159

Fig_S5_wraf159

Fig_S6_wraf159

Fig_S7_wraf159

Fig_S8_wraf159

Data_S1_wraf159

Data_S2_wraf159

Data_S3_wraf159

Data_S4_wraf159

Data_S5_wraf159

LGT_supplementary_ISME_250729_wraf159
