# Supplementary material for: Net rate of lateral gene transfer in marine prokaryoplankton
Source: ISME J. 2025 Sep 5;19(1):wraf159. doi: 10.1093/ismejo/wraf159 (PMC12416821; doi:10.1093/ismejo/wraf159)
Supplement: Fig_S1_wraf159 [file fig_s1_wraf159.pdf]

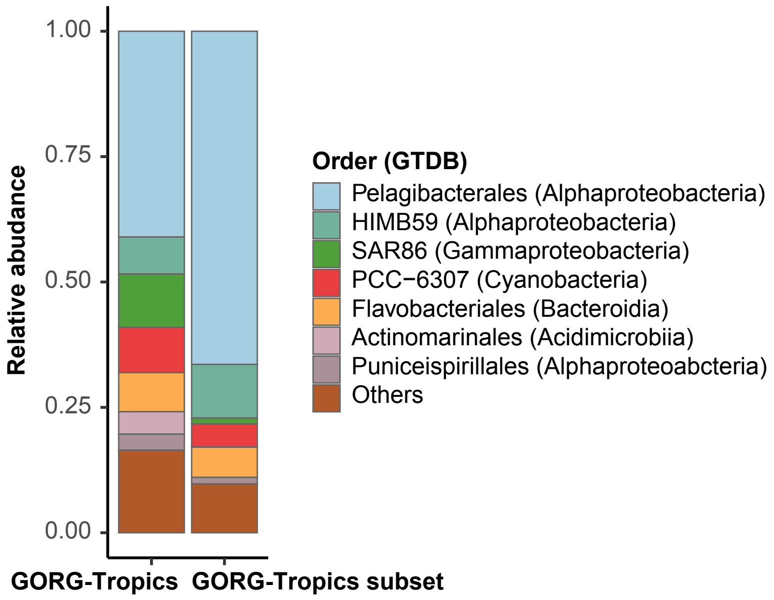

Fig. S1. Taxonomic composition of SAGs selected for this study, compared to the entire GORG-Tropics dataset.
