# Supplementary material for: Net rate of lateral gene transfer in marine prokaryoplankton
Source: ISME J. 2025 Sep 5;19(1):wraf159. doi: 10.1093/ismejo/wraf159 (PMC12416821; doi:10.1093/ismejo/wraf159)
Supplement: Fig_S2_wraf159 [file fig_s2_wraf159.pdf]

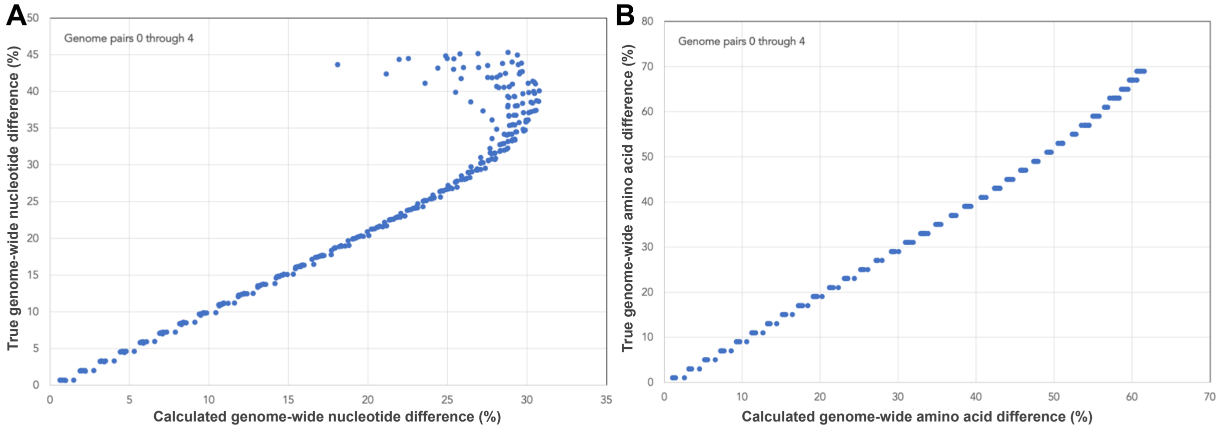

Fig. S2. Relationships between the estimated and true NDgenome (A) and AADgenome (B) in a simulated set of microbial genomes. Evolutionary divergence was emulated by *in silico* point mutation in four pairs of SAGs with near-identical genomes. No LGT was introduced in this simulation.
