# Supplementary material for: Net rate of lateral gene transfer in marine prokaryoplankton
Source: ISME J. 2025 Sep 5;19(1):wraf159. doi: 10.1093/ismejo/wraf159 (PMC12416821; doi:10.1093/ismejo/wraf159)
Supplement: Fig_S3_wraf159 [file fig_s3_wraf159.pdf]

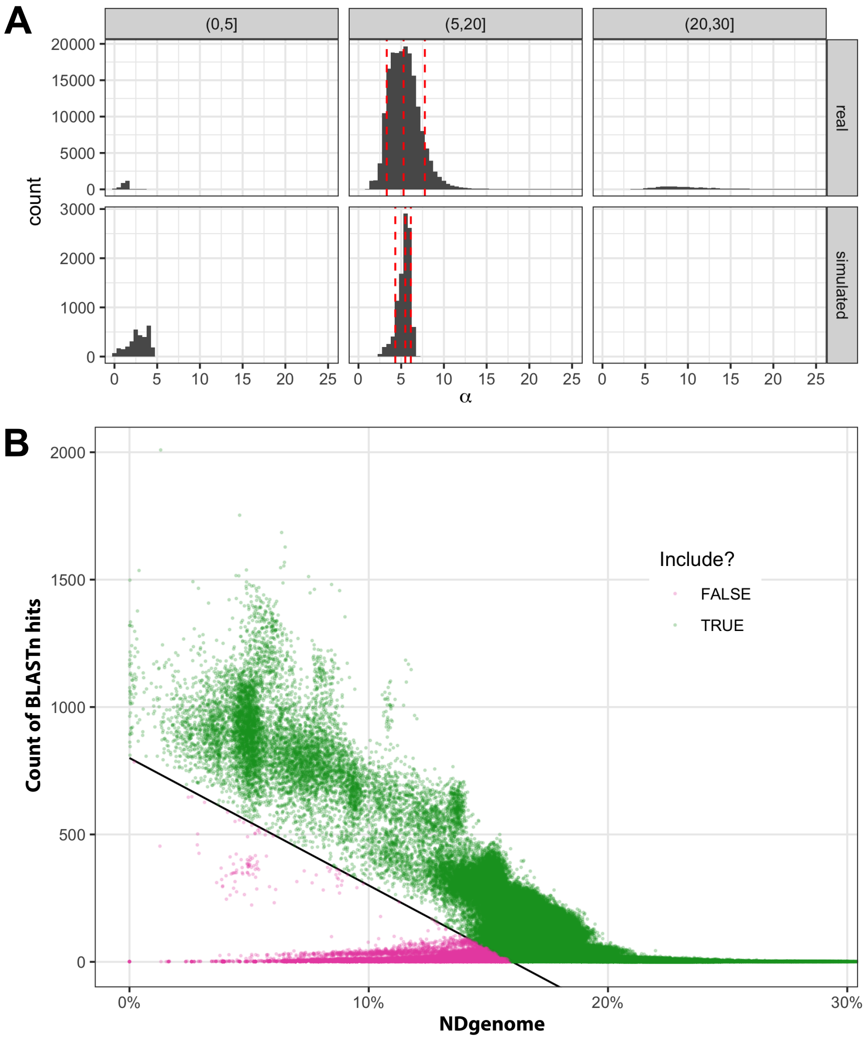

Fig. S3. Determination of the shape parameter α, the inverse of the variance of one-centered substitution rate multipliers across genes. (A) Distribution of α estimated for pairs of genomes in the GORG-Tropics (top) and a simulated, LGT-free dataset (bottom). We divided the set of genome pairs by their estimated NDgenome into three groups (thresholds set at 5% and 20% NDgenome). Genome pairs with <5% or >20% NDgenome had outlier estimates of α and thus were excluded. Quantiles of the distribution in the middle panel (3.31, 5.28, and 7.77) are shown as dotted lines. **(B)** Correlation between the count of BLASTn hits and NDgenome in pairs of GORG-Tropics genomes. Outliers below the shown line were excluded from α estimates.
