# Supplementary material for: Net rate of lateral gene transfer in marine prokaryoplankton
Source: ISME J. 2025 Sep 5;19(1):wraf159. doi: 10.1093/ismejo/wraf159 (PMC12416821; doi:10.1093/ismejo/wraf159)
Supplement: Fig_S4_wraf159 [file fig_s4_wraf159.pdf]

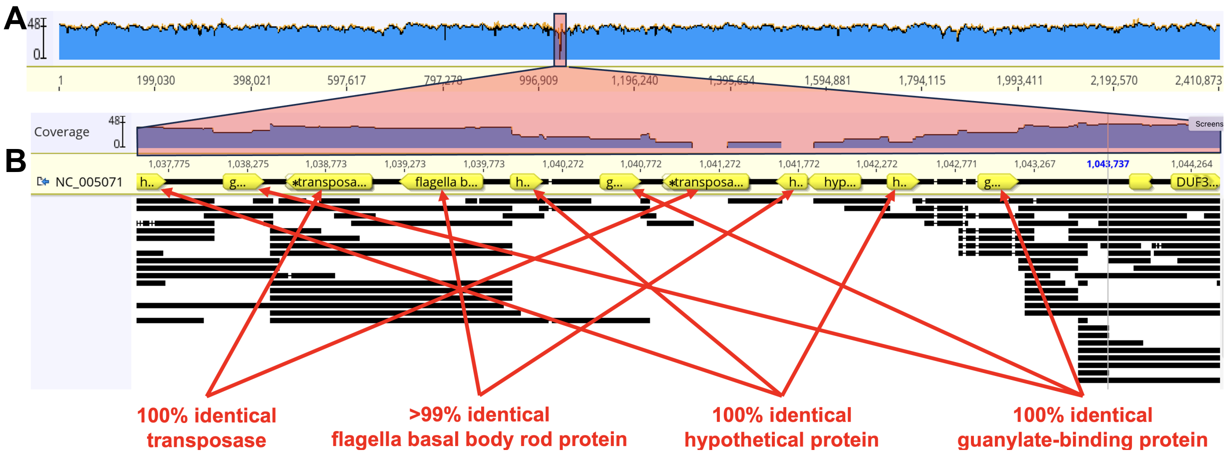

Fig. S4. Alignments of Prochlorococcus marinus MIT9313 SAG contigs on this strain's reference genome. (**A**) Coverage depth over the entire length of the genome and a highlighted genome region with reduced SAG representation. (**B**) Gene content and alignments of individual contigs in the highlighted genome region. A total of 50 SAGs of a monoclonal culture of *Prochlorococcus marinus* MIT9313 were generated and sequenced using the same protocols as in the production of GORG-Tropics dataset, as reported previously {Becraft, 2021 #7736}. The obtained contigs were mapped on the reference genome (RefSeq #NC005071) with Geneious v. 11.0.20.1 using default settings. The mapping results indicate a highly even genome coverage (**A**), with the region highlighted in **B** being the only region of MIT9313 with reduced coverage. This region contains multiple repeats, which likely led to the fragmentation of de novo genome assemblies. Importantly, individual genes of this repeat region are well represented in *Prochlorococcus marinus* MIT9313 SAGs and therefore do not impact GETS gene share estimates, which take into account only gene's presence/absence and not the number of its copies in a genome.
