# Supplementary material for: Net rate of lateral gene transfer in marine prokaryoplankton
Source: ISME J. 2025 Sep 5;19(1):wraf159. doi: 10.1093/ismejo/wraf159 (PMC12416821; doi:10.1093/ismejo/wraf159)
Supplement: Fig_S5_wraf159 [file fig_s5_wraf159.pdf]

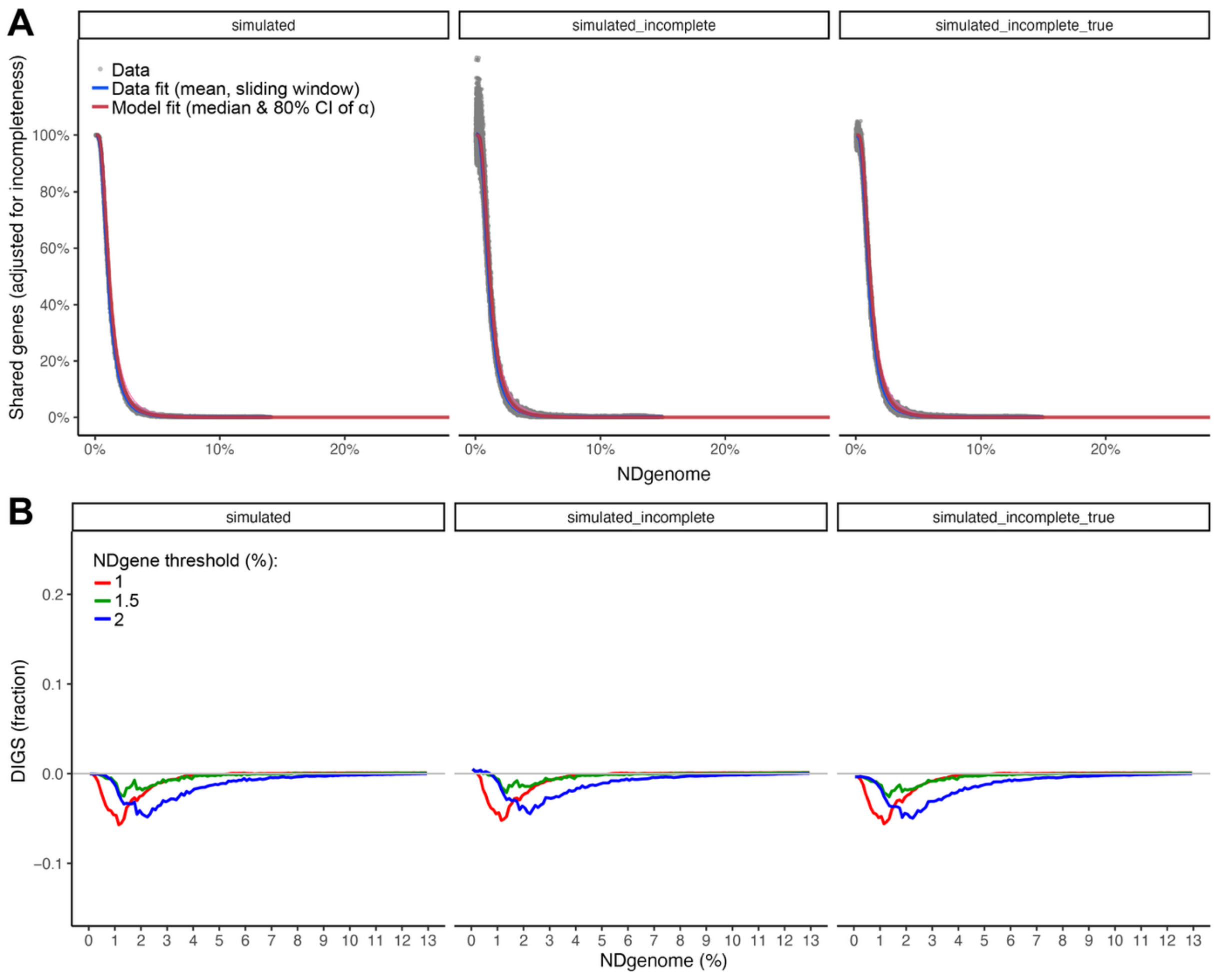

Fig. S5. Validation of the Discrepancy in Gene Share (DIGS) approach with genomes that were evolved *in silico* without LGT. (A) Observed and model-predicted fraction of shared genes with <1% NDgene in pairs of genomes along the gradient of NDgenome. The three panels show results from simulations with or without added incompleteness by removing genome chunks. Let the checkM-based completeness of a genome before and after removing chunks be c0 and c1, respectively, and the portion of a genome removed be p. We correct for incompleteness using 1- c1/c0 (simulated_incomplete) or p (simulated_incomplete_true). **(B)** Relationship between NDgenome and DIGS.
