# Supplementary material for: Net rate of lateral gene transfer in marine prokaryoplankton
Source: ISME J. 2025 Sep 5;19(1):wraf159. doi: 10.1093/ismejo/wraf159 (PMC12416821; doi:10.1093/ismejo/wraf159)
Supplement: Fig_S6_wraf159 [file fig_s6_wraf159.pdf]

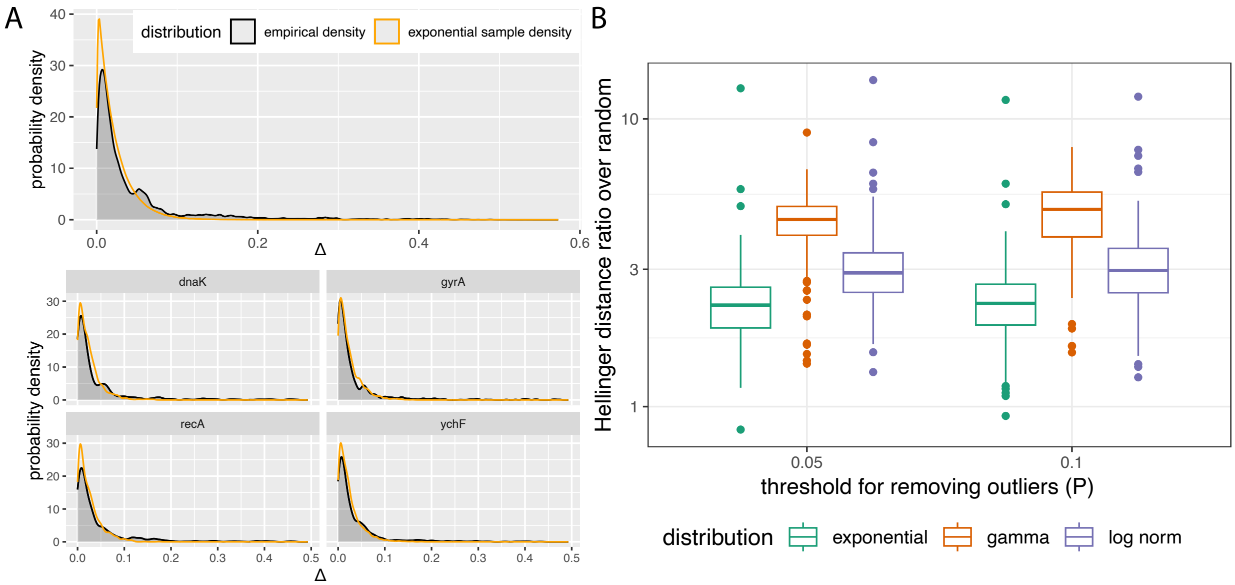

Fig. S6. Justification for statistical tests used to quantify deviation between gene trees and AADgenome values. (A) The empirical distribution of Δ for all genes (top) and four select genes, compared to Exponential distribution. We generally see a good fit. **(B)** Exponential is a much better fit to the real data than Gamma and LogNormal distributions, despite the latter distributions having two instead of one parameter. We compute the Hellinger distance H^* between the empirical distribution of the n available Δ values and a random sample of n points from Exponential, Gamma, or LogNorm distributions with estimated parameters, excluding outliers defined as those with CDF above $1-P$ for $P=0.05$ or $P=0.1$. We also compute the Hellinger distance between 1000 replicate samples from each of those distributions (each with size n) and average them to get H_0. We show the distribution of ratio H^*/H_0 across all genes. Δ values on real data (without outliers) are more similar to Exponential than alternative distributions.
