# Supplementary material for: Net rate of lateral gene transfer in marine prokaryoplankton
Source: ISME J. 2025 Sep 5;19(1):wraf159. doi: 10.1093/ismejo/wraf159 (PMC12416821; doi:10.1093/ismejo/wraf159)
Supplement: Fig_S7_wraf159 [file fig_s7_wraf159.pdf]

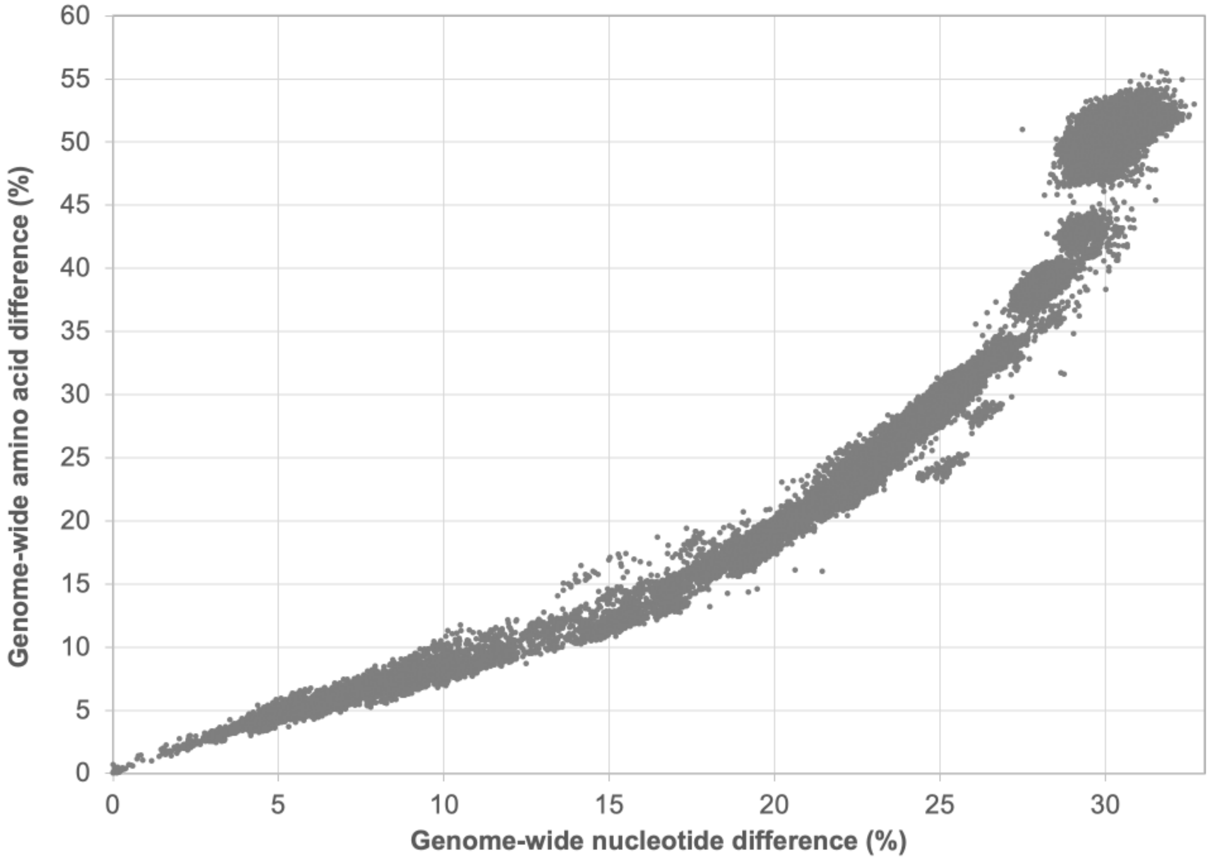

Fig. S7. Relationship between NDgenome and AADgenome. Included are 861 GORG-Tropics SAG assemblies with ≥80% estimated genome completion that contain 16S rRNA genes.
