# Supplementary material for: Net rate of lateral gene transfer in marine prokaryoplankton
Source: ISME J. 2025 Sep 5;19(1):wraf159. doi: 10.1093/ismejo/wraf159 (PMC12416821; doi:10.1093/ismejo/wraf159)
Supplement: Fig_S8_wraf159 [file fig_s8_wraf159.pdf]

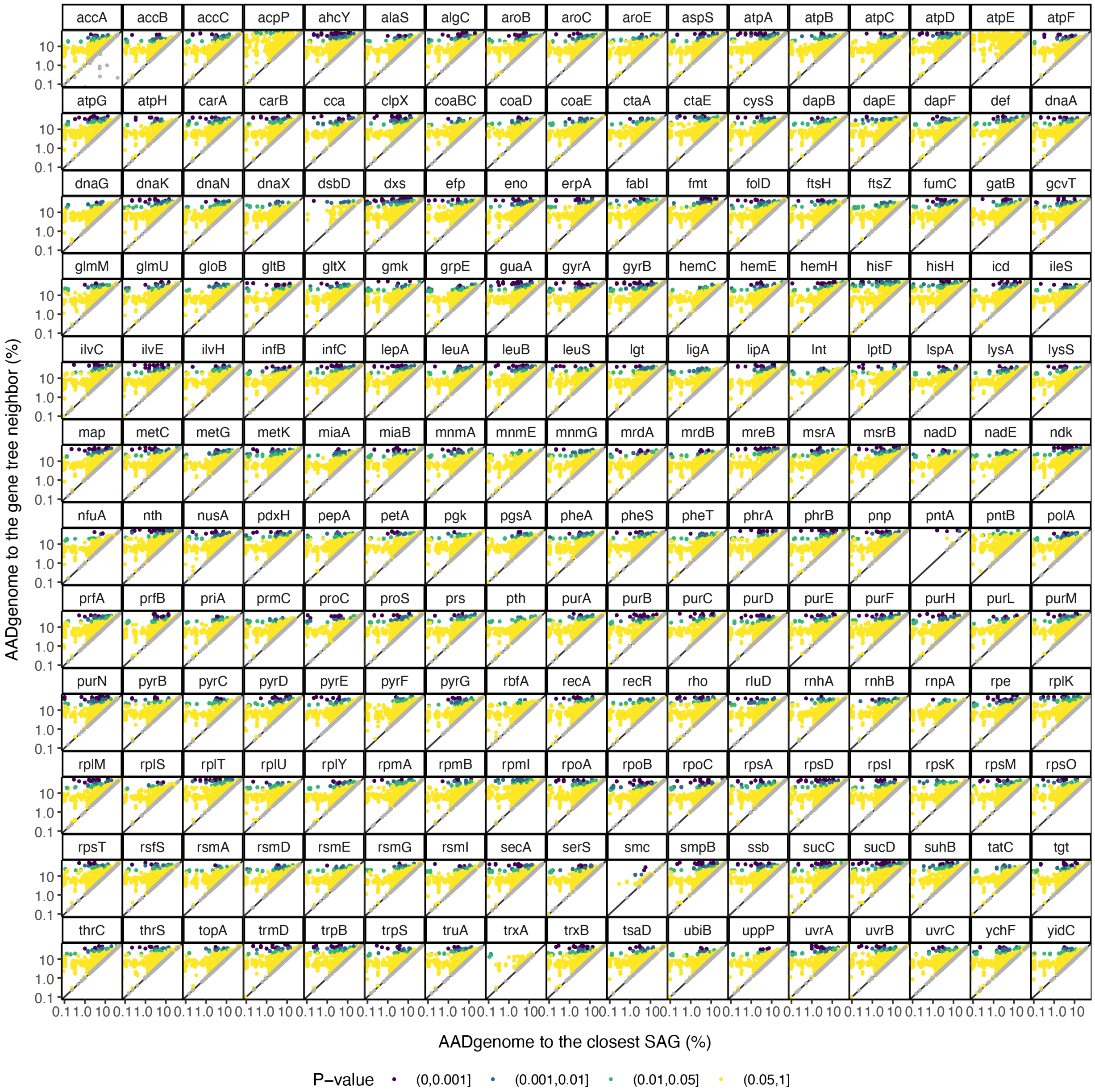

Fig. S8. Incongruencies between gene family trees and AADgenome. Outliers from the 1:1 relationship between the minimum AADgenome across all genomes (x-axis) and the AADgenome computed among the three closest neighbors on the gene tree (y-axis) indicate potential LGT. This analysis was performed on 204 protein-coding genes that were found in the largest number of GORG-Tropics SAGs and had only one or very few copies per genome (median, mean, max copy number: 1, 1.04, 4). Multi-copies were treated separately. The P-values indicate statistically significant deviation from 1:1 line by a parametric test. Data points where the minimum AADgenome across the genomes equaled the AADgenome to the three closest neighbors (i.e., x = y) were excluded from the outlier detection analysis and are shown in grey.
