# Supplementary material for: Net rate of lateral gene transfer in marine prokaryoplankton
Source: ISME J. 2025 Sep 5;19(1):wraf159. doi: 10.1093/ismejo/wraf159 (PMC12416821; doi:10.1093/ismejo/wraf159)
Supplement: LGT_supplementary_ISME_250729_wraf159 [file lgt_supplementary_isme_250729_wraf159.pdf]

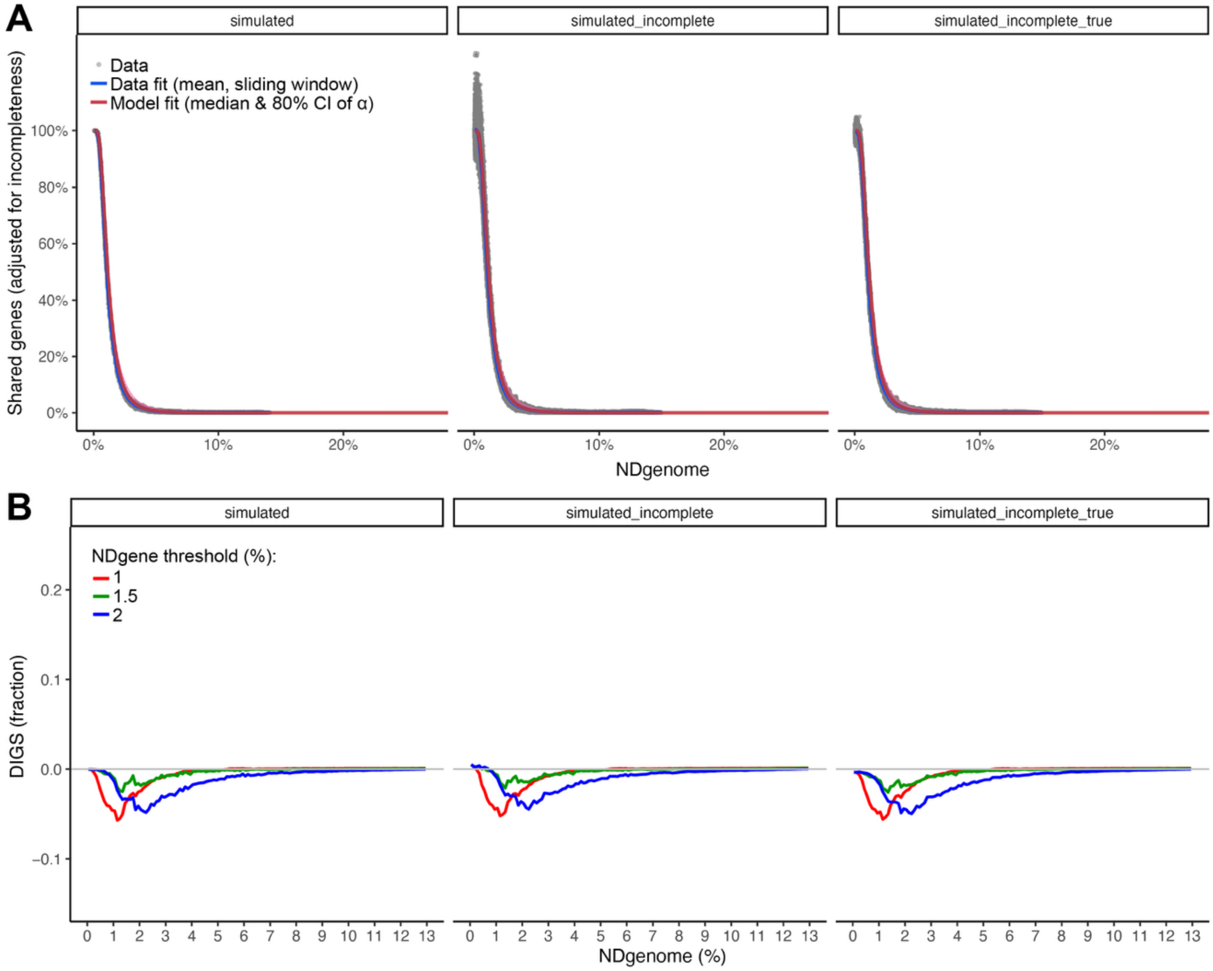

Fig. S1. Validation of the Discrepancy in Gene Share (DIGS) approach with genomes that were evolved *in silico* without LGT. (A) Observed and model-predicted fraction of shared genes with <1% NDgene in pairs of genomes along the gradient of NDgenome. The three panels show results from simulations with or without added incompleteness by removing genome chunks. Let the checkM-based completeness of a genome before and after removing chunks be c0 and c1, respectively, and the portion of a genome removed be p. We correct for incompleteness using 1- c1/c0 (simulated_incomplete) or p (simulated_incomplete_true). **(B)** Relationship between NDgenome and DIGS.

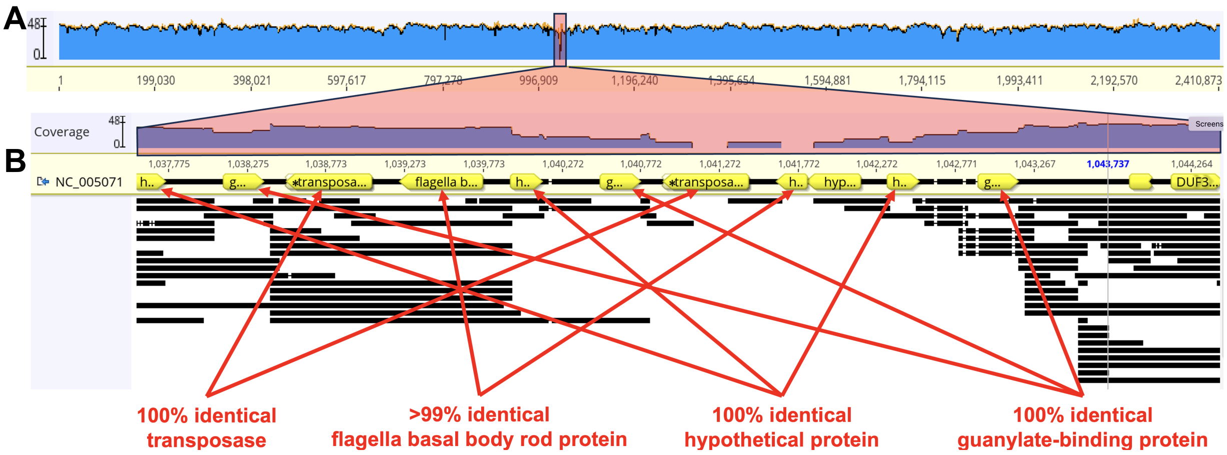

Fig. S2. Alignments of *Prochlorococcus marinus* MIT9313 SAG contigs on this strain's reference genome. (A) Coverage depth over the entire length of the genome and a highlighted genome region with reduced SAG representation. **(B)** Gene content and alignments of individual contigs in the highlighted genome region. A total of 50 SAGs of a monoclonal culture of *Prochlorococcus marinus* MIT9313 were generated and sequenced using the same protocols as in the production of GORG-Tropics dataset, as reported previously {Becraft, 2021 #7736}. The obtained contigs were mapped on the reference genome (RefSeq #NC005071) with Geneious v. 11.0.20.1 using default settings. The mapping results indicate a highly even genome coverage (**A**), with the region highlighted in **B** being the only region of MIT9313 with reduced coverage. This region contains multiple repeats, which likely led to the fragmentation of *de novo* genome assemblies. Importantly, individual genes of this repeat region are well represented in *Prochlorococcus marinus* MIT9313 SAGs and therefore do not impact GETS gene share estimates, which take into account only gene's presence/absence and not the number of its copies in a genome.

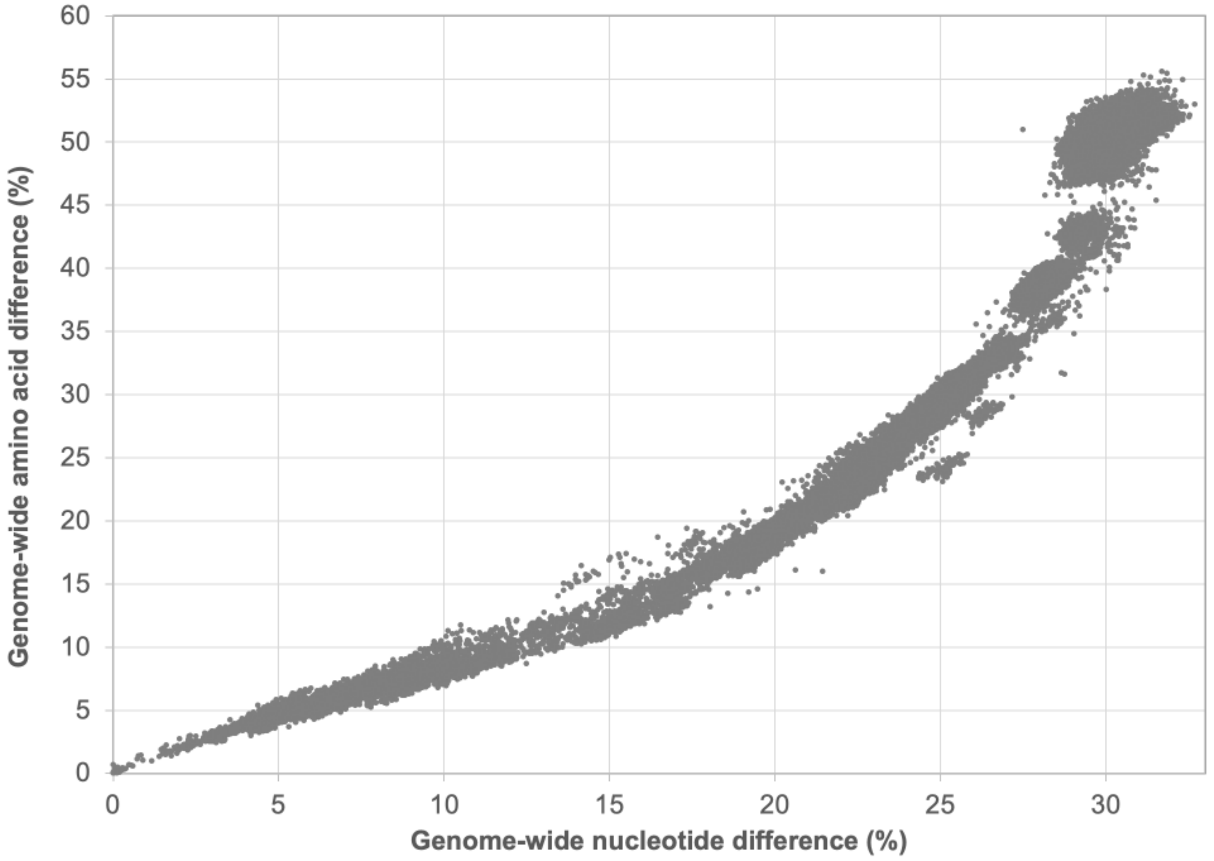

Fig. S3. Relationship between NDgenome and AADgenome. Included are 861 GORG-Tropics SAG assemblies with ≥80% estimated genome completion that contain 16S rRNA genes.

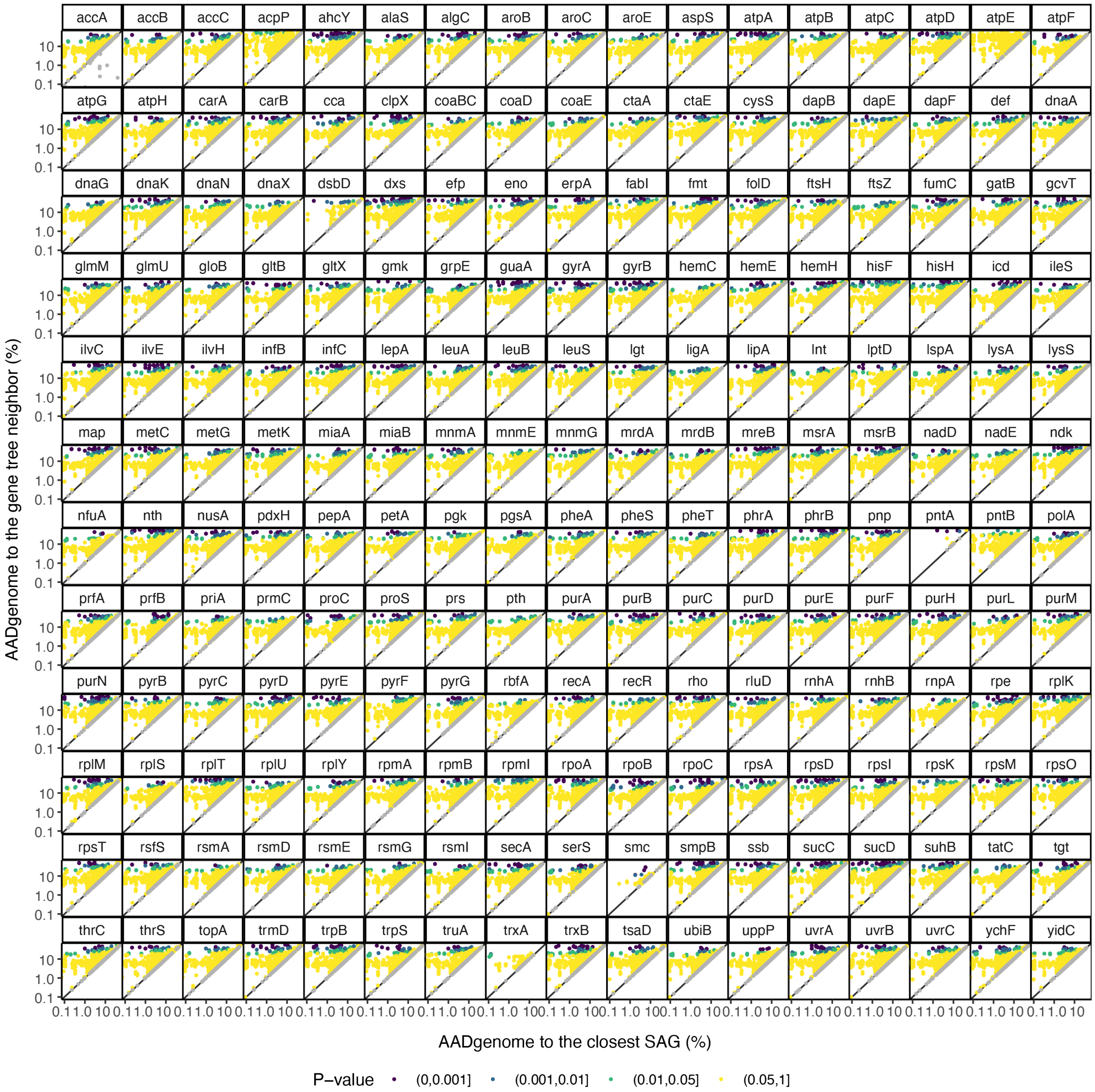

Fig. S4. Incongruencies between gene family trees and AADgenome. Outliers from the 1:1 relationship between the minimum AADgenome across all genomes (x-axis) and the AADgenome computed among the three closest neighbors on the gene tree (y-axis) indicate potential LGT. This analysis was performed on 204 protein-coding genes that were found in the largest number of GORG-Tropics SAGs and had only one or very few copies per genome (median, mean, max copy number: 1, 1.04, 4). Multi-copies were treated separately. The P-values indicate statistically significant deviation from 1:1 line by a parametric test. Data points where the minimum AADgenome across the genomes equaled the AADgenome to the three closest neighbors (i.e., x = y) were excluded from the outlier detection analysis and are shown in grey.

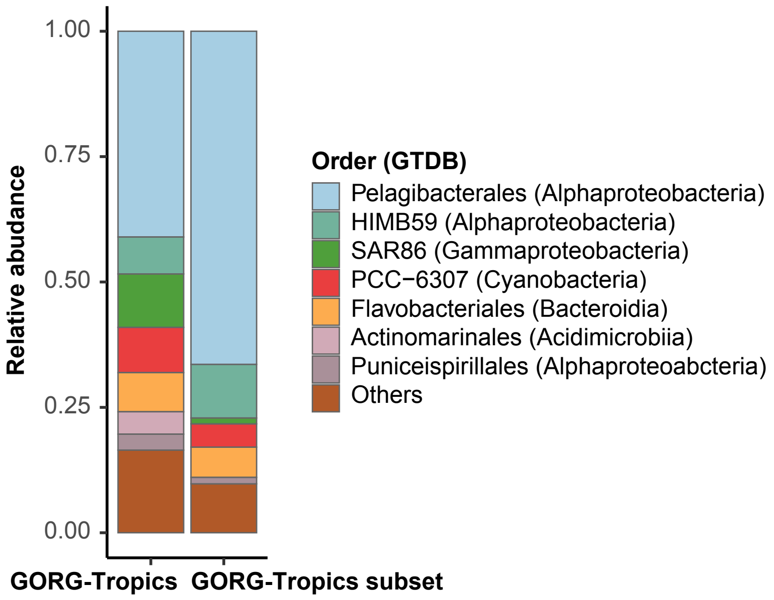

Fig. S5. Taxonomic composition of SAGs selected for this study, compared to the entire GORG-Tropics dataset.

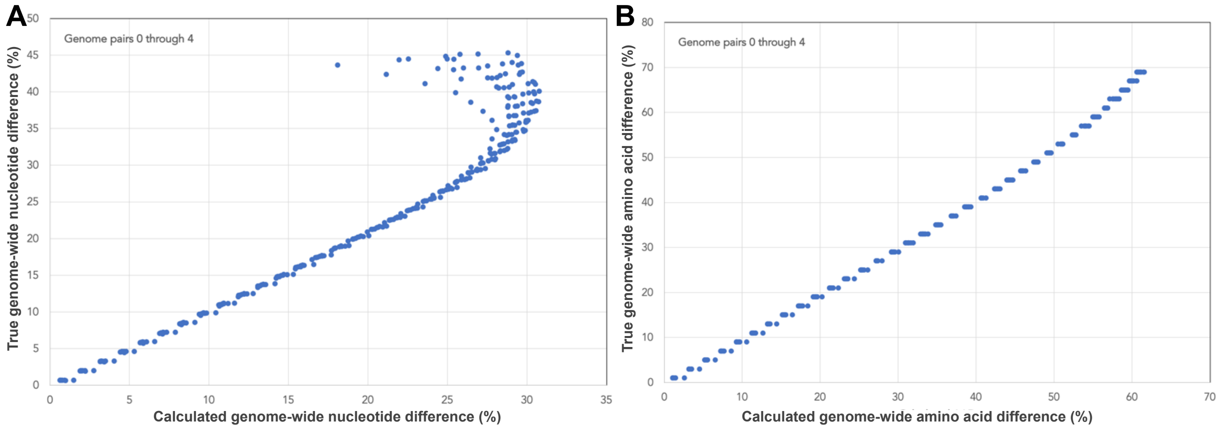

Fig. S6. Relationships between the estimated and true NDgenome (A) and AADgenome (B) in a simulated set of microbial genomes. Evolutionary divergence was emulated by *in silico* point mutation in four pairs of SAGs with near-identical genomes. No LGT was introduced in this simulation.

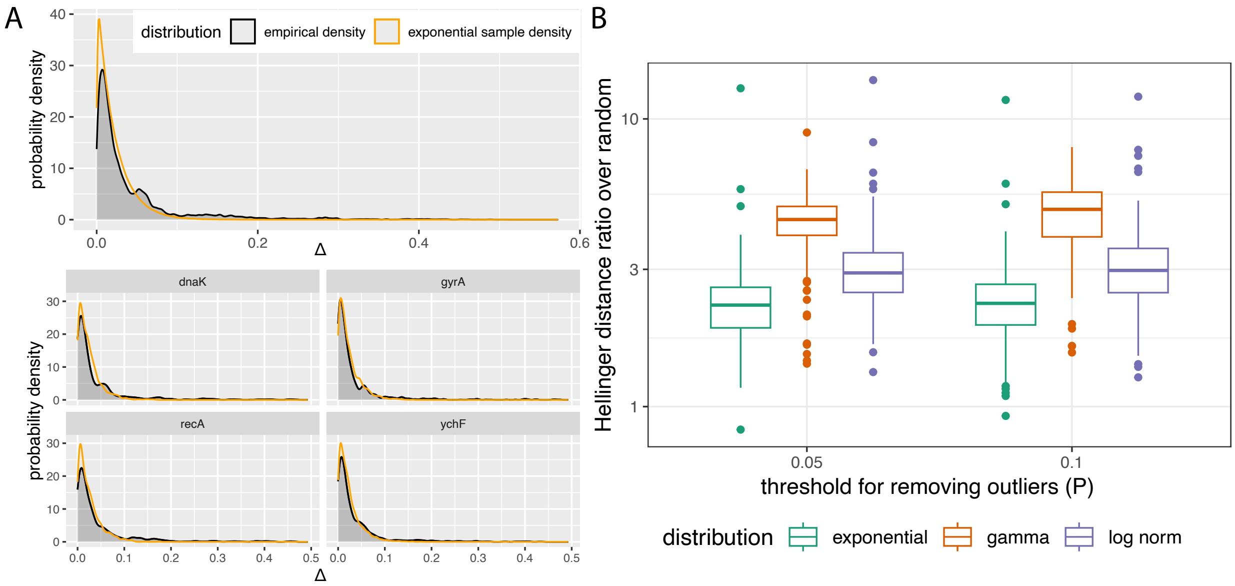

Fig. S7. Justification for statistical tests used to quantify deviation between gene trees and AADgenome values. (A) The empirical distribution of Δ for all genes (top) and four select genes, compared to Exponential distribution. We generally see a good fit. **(B)** Exponential is a much better fit to the real data than Gamma and LogNormal distributions, despite the latter distributions having two instead of one parameter. We compute the Hellinger distance H^* between the empirical distribution of the n available Δ values and a random sample of n points from Exponential, Gamma, or LogNorm distributions with estimated parameters, excluding outliers defined as those with CDF above $1-P$ for $P=0.05$ or $P=0.1$. We also compute the Hellinger distance between 1000 replicate samples from each of those distributions (each with size n) and average them to get H_0. We show the distribution of ratio H^*/H_0 across all genes. Δ values on real data (without outliers) are more similar to Exponential than alternative distributions.

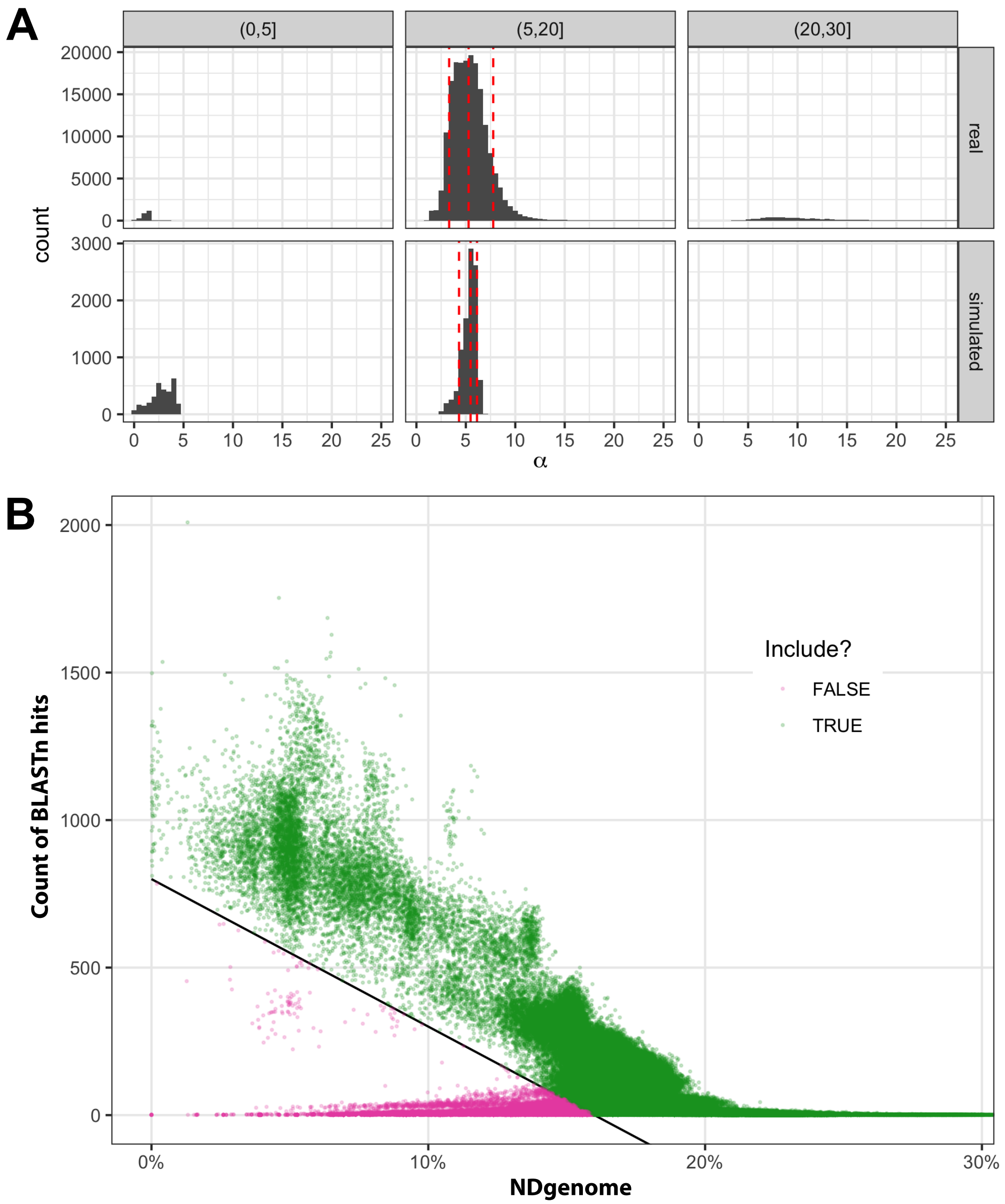

Fig. S8. Determination of the shape parameter α, the inverse of the variance of one-centered substitution rate multipliers across genes. (A) Distribution of α estimated for pairs of genomes in the GORG-Tropics (top) and a simulated, LGT-free dataset (bottom). We divided the set of genome pairs by their estimated NDgenome into three groups (thresholds set at 5% and 20% NDgenome). Genome pairs with <5% or >20% NDgenome had outlier estimates of α and thus were excluded. Quantiles of the distribution in the middle panel (3.31, 5.28, and 7.77) are shown as dotted lines. **(B)** Correlation between the count of BLASTn hits and NDgenome in pairs of GORG-Tropics genomes. Outliers below the shown line were excluded from α estimates.

Data S1. Characteristics of GORG-Tropics SAGs selected for this study.

Data S2. Pairwise comparisons of GORG-Tropics SAGs selected for this study.

Data S3. Genes with NDgene <2% in pairs of genomes from different taxonomic orders.

Data S4. Genome pairs used in LGT-free evolution simulation for the evaluation of NDgenome and AADgenome estimate accuracy.

Data S5. Genome pairs used in LGT-free evolution simulation for the evaluation of DIGS estimate accuracy.
